# New monoclonal antibodies that recognize an unglycosylated, conserved, extracellular region of CD44 *in vitro* and *in vivo*, and can block tumorigenesis

**DOI:** 10.1371/journal.pone.0250175

**Published:** 2021-04-23

**Authors:** Daniel F. Lusche, Deborah J. Wessels, Ryan J. Reis, Cristopher C. Forrest, Alexis R. Thumann, David R. Soll

**Affiliations:** Developmental Studies Hybridoma Bank, Department of Biology, University of Iowa, Iowa City, Iowa, United States of America; Duke University School of Medicine, UNITED STATES

## Abstract

CD44 is a transmembrane glycoprotein that binds to hyaluronic acid, plays roles in a number of cellular processes and is expressed in a variety of cell types. It is up-regulated in stem cells and cancer. Anti-CD44 monoclonal antibodies affect cell motility and aggregation, and repress tumorigenesis and metastasis. Here we describe four new anti-CD44 monoclonal antibodies originating from B cells of a mouse injected with a plasmid expressing CD44 isoform 12. The four monoclonal antibodies bind to the terminal, extracellular, conserved domain of CD44 isoforms. Based on differences in western blot patterns of cancer cell lysates, the four anti-CD44 mAbs separated into three distinct categories that include P4G9, P3D2, and P3A7, and P3G4. Spot assay analysis with peptides generated in *Escherichia coli* support the conclusion that the monoclonal antibodies recognize unglycosylated sequences in the N-terminal conserved region between amino acid 21–220, and analyses with a peptide generated in human embryonic kidney 293 cells, demonstrate that these monoclonal antibodies bind to these peptides only after deglycosylation. Western blots with lysates from three cancer cell lines demonstrate that several CD44 isoforms are unglycosylated in the anti-CD44 target regions. The potential utility of the monoclonal antibodies in blocking tumorigenesis was tested by co-injection of cells of the breast cancer-derived tumorigenic cell line MDA-MB-231 with the anti-CD44 monoclonal antibody P3D2 into the mammary fat pads of mice. All five control mice injected with MDA-MB-231 cells plus anti-IgG formed palpable tumors, while only one of the six test mice injected with MDA-MB-231 cells plus P3D2 formed a tiny tumor, while the remaining five were tumor-free, indicating that the four anti-CD44 mAbs may be useful therapeutically.

## Introduction

CD44, a transmembrane glycoprotein with a large extracellular domain [1–5], was originally identified as a receptor for the extracellular matrix molecule hyaluronic acid [6–9]. Subsequently, it was shown that CD44 played a role in a number of cellular processes related to adhesion and cell motility [2, 10–13], in some cases in the absence of hyaluronic acid [14, 15]. CD44 is expressed in a variety of cell types [16–18], and has been shown to be up-regulated in stem cells [19–22] as well as select carcinomas [23–31]. Anti-CD44 monoclonal antibodies (mAbs) have been shown to block the formation of subcutaneous tumors and to repress metastasis in mice [32, 33], and down regulation of CD44 in cancer cells has been shown to reduce stem cell-associated traits [34–36]. Moreover, anti-CD44 mAbs have been shown to affect cancer cell motility and aggregation of breast tumor and melanoma cell lines in a 3D Matrigel model, in the apparent absence of hyaluronic acid (HA) [14, 15].

For investigating the role of CD44 in tumorigenesis and metastasis, there are over 140 commercially available anti-CD44 mAbs (S1 Table), the majority generated against peptides representing conserved regions of the protein. There are, however, problems in studying the role of CD44 in tumorigenesis and metastasis with mAbs, given the large number of isoforms resulting from alternative splicing and secondary modification, most notably glycosylation (https://www.ncbinlm.nih.gov/protein; https://www.uniprot.org; HGNC1681 at HUGO; https://www.genenames.org) [5, 37–42]. Through alternative splicing alone, at least 38 CD44 mRNAs have been identified, and by protein separation methods, at least 21 isoforms [42]. Many of these isoforms probably play specialized roles in different cellular functions and are cell type-specific. In tumorigenesis and metastasis, the different isoforms may play roles that are specific to different cancers. Although there are more than 140 commercially available antibodies (S1 Table), many either represent the same original mAbs or target the same region of the CD44 protein. Given the variety of functions, combined with the potential number of isoforms of CD44 expressed in cancer cells, variations in the heavy chain and light chain sequences of the different anti-CD44 mAbs, as well as variations in the targeted antigen sequences, it is unlikely that the number of available mAbs is sufficient. Indeed, the mAbs with highest efficacy and specificity and with optimum therapeutic value may have been missed. For that reason, we have begun to generate and characterize new anti-CD44 mAbs. Here, we describe four anti-CD44 mAbs generated against a recombinant CD44 generated by a plasmid expressing CD44isf(isoform)12 injected into a mouse. CD44isf12 lacks the entire extracellular variable region. CD44isf12, is upregulated in breast cancer [43, 44], and has been implicated in the epithelial-mesenchymal transition (EMT) and tumor progression in mice [45]. CD44isf12 retains the complete extracellular conserved regions that contains the hyaluronidase binding domain, the transmembrane domain and the cytoplasmic tail [13]. Because the CD44 antigen used in the immunization was generated in the mouse, the plasmid expressed a glycosylated CD44 protein, but glycosylation was not necessarily like that of native CD44 proteins formed in human cancer cells. We selected four of ten anti-CD44isf12 mAbs for further analysis, based on their rate of hybridoma growth, IgG subtype, and intensity of ELISA selection. The four anti-CD44 mAbs, produced by the four hybridomas, P4G9, P3D2, P3A7 and P3G4, stained fixed cells of three different tumorigenic cell lines(breast, melanoma, glioblastoma) and a nontumorigenic breast cell line. Staining by the four mAbs occurred at the plasma membrane. All four mAbs recognized multiple CD44 isoforms in lysates of the three cell lines by western blot analyses, and bound to recombinant peptides containing the unglycosylated N-terminal conserved region of CD44, generated in *Escherichia coli (E*. *coli*). The mAbs did not bind to a similar peptide generated in the human embryonic kidney cell line HEK293, which was glycosylated. However, if this HEK293 recombinant protein, representing amino acids (aa) 1 through 220 (the entire N-terminal extracellular conserved region of CD44), was deglycosylated with neuramidase and PNGaseF, all four mAbs bound to it, indicating that the mAbs recognized only the unglycosylated peptide. This was verified by a spot assay using an unglycosylated peptide generated in *E*. *coli* with the same amino acid sequence as the HEK293-generated peptide. Since the four mAbs stained fixed cells of three tumorigenic cell lines (MDA-MB-231, HTB-66, U87) and bound to multiple isoforms in western blots of lysates of the three cell lines, it seems reasonable to conclude that multiple CD44 isoforms that are unglycosylated in the anti-CD44 target regions, are expressed in the unglycosylated form three cell lines. Finally, we demonstrate that the mAb P3D2 inhibits tumorigenesis by cells of the breast cancer cell line in mammary fat pads of mice, suggesting therapeutic value.

## Material and methods

### Antibodies and peptides

The anti-c-MYC mAb 9E10, developed by M.J. Bishop, was obtained from the Developmental Studies Hybridoma Bank (DSHB) (http://dshb.biology.uiowa.edu/). The affinity purified goat anti-mouse IgG1 (Cat.# A90-105A) was obtained from Bethyl Laboratories. The recombinant (p1) CD44 80 kDa CD44-6X Histidine-Sumo fusion protein corresponding to aas 21–606 of CD44 isoform 4 (Cat.# LS-G20578), and the recombinant (p3) CD44-6X Histidine fusion protein corresponding to aas 21–220 (Cat.# LS-G26414) were obtained from LifeSpan BioScience, Inc. The (p2) CD44 histidine (HIS) fusion protein, corresponding to aa 28–380 of the CD44 isoform 4 (Cat.# AG7633) was obtained from Proteintech. The recombinant n-terminal (p4) 23.4 kDa CD44-6X Histidine fusion protein corresponding to aas 1–220 (Cat.# 12211-H08H) was obtained from Sinobiologicals. The Multiple Tag protein containing 6X Histidine and the V5 tag (Cat.# M0101) was obtained from GenScript.

### Cell culture

The cell lines used in this study were obtained from the American Type Culture Collection (ATCC). The nontumorigenic cell line MCF-7 was cultured in Eagles Minimal Medium (MEM) (Cat.# 11095–080; ThermoFisher Scientific) supplemented with 10% FBS (Cat.# S11150; Atlanta Biologicals), 10 μg/ml insulin (Cat.# I-9728; Millipore-Sigma) and 1% penicillin- streptomycin mixture (Cat.# 15140122; ThermoFisher Scientific). The tumorigenic MDA-MB-231 (MB-231) cell line, was cultured in DMEM/F12 medium (Cat.# 11320033; ThermoFisher Scientific), supplemented with 5% horse serum (Cat.# 16050122; ThermoFisher Scientific), 1% penicillin-streptomycin mixture, 20 ng/ml of human recombinant EGF (Cat.# E-9644), 10 μg/ml insulin, 0.5 μg/ml hydrocortisone (Cat.# H-0135) and 0.1 μg/ml cholera toxin (Cat.# C-8052) all obtained from Millipore-Sigma. Cells of the HTB-66 and U87 cell lines were cultured in MEM supplemented with 10% FBS, 1% sodium pyruvate (Cat.# 11360070) and 1% nonessential amino acids (Cat.# 11140050), both from ThermoFisher Scientific. Hybridomas were cultured in Hyclone ADCF mAb medium (Cat.# SH30349.02; GE Healthcare), containing 1% glutamax (Cat.# 35050061), 1% sodium pyruvate, 1% penicillin streptomycin (all ThermoFisher Scientific) and 0.1% gentamycin (Cat.# IB02030, IBI Scientific), at 37°C in 7.5% CO_2_. Supernatants were harvested from hybridoma cultures that had reached concentrations of 1.5 to 2.0x10^6^ cells per ml.

### RNA isolation

Total cell RNA was isolated as previously described [46]. In brief, cells in tissue culture flasks were washed with phosphate buffered saline (PBS), pH7.2, and 1 ml of Trizol (Cat.# 15596026; ThermoFisher Scientific) added. The preparation was incubated for 5 min. 200 μl of chloroform was added, incubated for 3 min and the mixture spun at 12000g for 20 min. The aqueous phase containing the RNA was precipitated with isopropanol, centrifuged at 12000g for 10 min., washed once with 75% ethanol and pelleted. The pellet was suspended in RNAase-free water. To eliminate residual genomic DNA, the RNA preparation was treated with gDNA wipeout buffer (Cat.# 205311; Qiagen).

### RT-PCR

RT-PCR was performed as previously described [46], with minor modifications. In brief, 1 μg of total RNA was subjected to RT-PCR using 3Ꞌ Primer rapid amplification of cDNA ends (RACE; Cat.# 18373–027; ThermoFisher Scientific). Reverse transcription was performed using the Adapter primer provided, which allows transcription of the poly(A) tail by Superscript II Reverse Transcriptase. The reaction, run at 42°C for 50 min, was terminated at 70°C for 15 min and by addition of 1 μl of RNaseH. 200 ng of cDNA was then used to amplify CD44 cDNAs, using the Expand Long Template Polymerase Kit (Cat.# 11681834001; Roche) and the primers CD44Fw 5Ꞌ and CD44Rv 5Ꞌ (S2 Table), corresponding to aa 353–361 (QNVDMKIGV) of CD44isf12. The PCR conditions were denaturation at 94°C for 2 minutes, 10 cycles with each at 94°C for 10 sec, 55°C for 30 sec, 68°C for 2.5 min, then 25 cycles with each at 94°C for 10 secs, 25 cycles at 55°C for 30 sec, 68°C for 2.5 min, progressively increased to 10 min. For amplification of the GAPDH, 100 ng of cDNA were generated with the primers GAPDH Fw 5Ꞌ and GAPDHRv 5Ꞌ (S2 Table).

### Cloning of CD44 cDNA fragments and generating expression plasmid

RT-PCR fragments were isolated using the Rapid Gel Isolation Kit (Cat.# 28704; Qiagen) and cloned using TOPO-pCR4.0 plasmid (Cat.# K457501; ThermoFisher). Plasmid purification was achieved using PureLink Quick Plasmid DNA Miniprep kit (Cat.# K210010; ThermoFisher). Sequencing of plasmid inserts was done at the Carver Center for Genomics at the department of biology at University of Iowa. The resulting sequencing plasmid pCR4.0-CD44isf12, which contained the fragment from HTB-66 cells, was used to the generate the expression plasmid.

### Creating the expression plasmid

The plasmid, pLX304-CD44, obtained from DNASU (https://dnasu.org/DNASU/), contained a CD44 fragment (2097 Bp) fused to a V5 epitope tag, under control of the CMV promoter. pLX304-CD44 was digested with BstXI (Cat.# RO113S; New England Biolabs) and BamH1 (Cat.# FD0054; ThermoFisher Scientific), and a 1436 Bp sequence including the variable region. The remaining pLX304 fragment was dephosphorylated, using artic alkaline phosphatase (Cat.# MO289S; New England Biolabs) and gel-purified. The plasmid, pCR4.0-CD44isf12, was then digested with BstXI and BamHI, to obtain a 422 Bp fragment that was subsequently gel purified. The two fragments were ligated using T4 Ligase (Cat.# 10481220001;Roche) and the resulting plasmid, pLX304isf12V5, which contained the CD44isf12 sequence fused in frame with a V5 tag, at the expression site, transformed into TOP10 cells (Cat.# C4040; Life Technologies) and sequenced. To test for expression, MB-231 cells were transformed with pLX304isf12V5 as previously described [46].

### Mouse immunization

**Fig 1:**
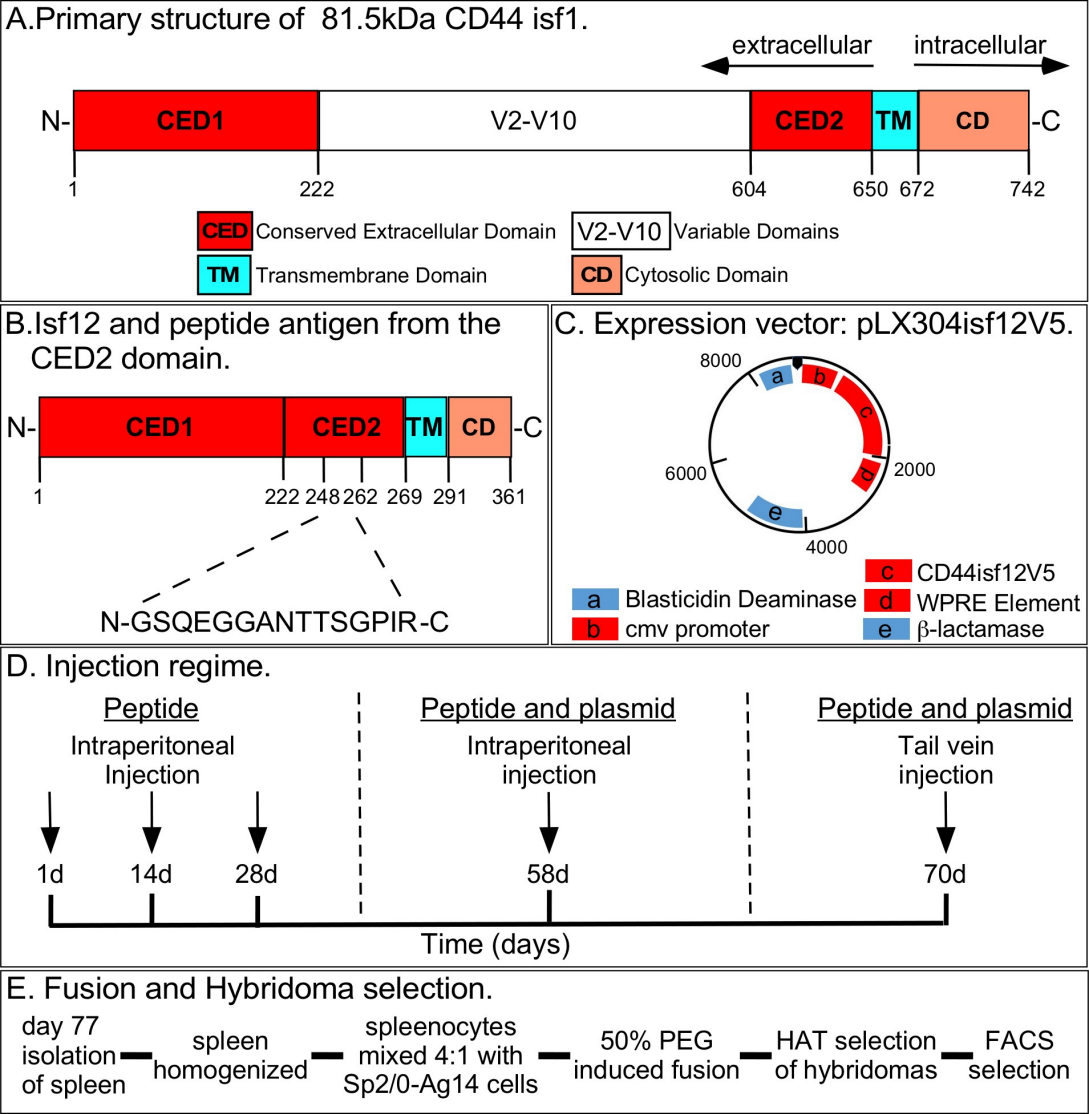
Primary structure of CD44isf1 and CD44isf12, the expression vector pLX304isf12V5, injection regime, and fusion and hybridoma selection. A. The primary structure of the largest CD44, CD44isf1, which contains the complete complement of variable regions. B. Primary structure of the smallest transmembrane CD44 isoform, CD44isf12, which lacks the entire variable domain, and the sequence of the CED2 domain representing the peptide used in the immunization regimes. C. The expression vector, pLX304isf12V5, used in the immunization regime. D. The mouse injection regime for generating anti-CD44 mAbs. E. Fusion and hybridoma selection regime for hybridomas producing anti-CD44 mAbs.

Mouse immunization (Fig 1D) was performed as approved under protocol 5121587 of the University of Iowa Institutional Animal Care and Use Committee (IACUC). All efforts were taken to minimize animal suffering. Balb/cAnNHsd mice (Cat.# 047; Envigo) were injected intraperitoneally (IP) with 50 μg of the CD44 peptide N-GSQEGGANTTSGPIR-C (>80% purity) (JPT Peptide Technologies) (Fig 1B) and 40 μl of Imject^TM^ Alum adjuvant (Cat.# 77161; ThermoFisher Scientific) at 1, 14 and 28 days. The peptide plus 5 μg of pX304-CD44isf12V5 were co-injected intraperitoneally at 58 days and in the tail vein at 70 days. The final intravascular co-injection included 50 μg of high molecular weight Vacci Grade Poly I:C (Cat.# teve-pic; InvivoGen) and 50 μg of anti-CD40 (Cat.# FGK4.5/FGK45; BioXCell) as adjuvant in a total volume of 200 μl. At 77 days, animals were euthanized, and spleens removed and homogenized.

### Hybridoma production and selection

Cell fusion was performed as previously described [47, 48], with minor modifications. Following euthanizing protocols approved by the Office of the Institutional Animal Use Committee, the spleen was removed, and splenocytes isolated and resuspended in Iscove’s modified Dulbecco’s medium (IMDM; Cat.# 12440053; ThermoFisher Scientific) supplemented with 1% glutamax, 1% sodium pyruvate, 0.1% penicillin streptomycin, and 0.1% gentamicin and 15% fetal bovine serum. Cells were then washed in serum-free IMDM medium. Sp2/0-Ag14 myeloma cells (Cat.# CRL-1581;ATCC) were also washed in serum free IMDM medium, passed through a 70 μm filter, and resuspended in serum-free IMDM. The nucleated splenocytes were then combined with Sp2/0-Ag14 myeloma cells at a ratio of 4:1 and pelleted. The pellet was resuspended in 0.5 ml of 50% (v/v) polyethylene glycol (PEG; Cat.# P7181; Millipore-Sigma)-IMDM medium, and 15 ml of prewarmed serum-free IMDM was added. The cell preparation was incubated at room temperature for 8 min without agitation, then at 37°C for 2 additional minutes, and pelleted. The pellet was resuspended in 30 ml of IMDM containing 15% fetal bovine serum, 20% IMDM medium conditioned by giant cell tumor (Cat.# TIB-223; ATCC), 0.1% sodium pyruvate, 0.1% glutamax and 50 μg/ml gentamicin sulfate. The cell suspension was then transferred to two T75 flasks and incubated at 37°C in 5% CO_2_ for 2 days. A 500 μl aliquot of 2X hypoxanthine–aminopterin–thymidine (HAT) (Cat.# HO262; Millipore-Sigma) medium was added to the flask, containing 5 ml growth medium, and the medium was exchanged every 3 days at 37°C in 5% CO_2_ in order to select for hybridomas. Upon 25% confluency, the cells were prepared for and sorted via fluorescent activated cell sorting (FACS) into 96 well plates.

### Transformation of MB-231 with pLX304isf12V5

To test expression of CD44isf12 in MB-231 cells, MB-231 cells were transformed using Fugene (Cat.# E2311; Promega) as described by the manufacturer [46]. In brief, 5x10^4^ cells in a 12 well tissue culture plate were grown overnight in regular growth media., then grown overnight in 500 μl Opti-MEM (Cat.# 31985062; ThermoFisher Scientific). A mixture of 2 μg of pLX304isf12V5plasmid/98 μl of Opti-MEM was incubated in the presence of 5 μl of Fugene and Opti-MEM in a total volume of 105 μl for 20 min. This mixture was then added to the cell culture plate, in 500 μl fresh growth media, and incubated for two days. Selection was started with 2 μg/ml Blasticidin (Cat.# ALX-380-089, Enzo Life Science) and subsequently increased to 10 μg/ml.

### FACS

HAT-selected hybridomas were washed once in serum-free IMDM medium, and the pellet resuspended in 1ml PBS, pH 7.2, and incubated with affinity-purified fluorescent anti-mouse IgG1 H+L Alexa Fluor 488 antibody (Cat.# AB_2338840; Jackson ImmunoResearch Laboratories. Inc.) at 1:10,000 dilution and propidium iodide (50 nM) (Cat.# P41701; Sigma). The antibody labeled hybridomas and unlabeled control hybridomas were filtered through a 70 μm mesh, and FACS performed at the University of Iowa Flow Cytometry Facility. IgG1 positive hybridomas were clonally plated into 96 well plates.

### mAb concentration and purification

mAb concentration and purification were performed as previously described [14, 15]. In brief, mAb supernatant was concentrated in VivaCell centrifugal filters with a 50 kDa cut off (Satorius, Stonehouse, UK). Concentrates were affinity purified using a Protein G HP spin trap column (Cat.# 28903A; GE Healthcare), desalted twice with a 4 ml Amicon Ultra-centrifugal filter (Cat.# UFC805024; EMD Millipore). The concentrations of purified mAbs were determined using a NanoDrop One UV-VIS 1000 Spectrophotmeter (ThermoFisher Scientific, Waltham, MA).

### Isotyping and ELISA

Isotypes of mAbs were assessed by using the IsoStrip, Mouse Monoclonal Antibody Isotyping Kit (Cat.# 11493027001; Roche). 10 μl of antibody concentrate was diluted 1:15 in PBS pH7.2 containing 1% BSA. A 150 μl aliquot was added to a tube containing lyophilized latex beads. After the beads were resuspended, the isotyping strip was placed in the preparation and the isotype identified. Enzyme-linked immunosorbent assay (ELISA) was performed as previously described with minor modifications [48]. One hundred ng of a recombinant CD44 (CD44 std.) 80 kDa protein (Cat.# BMS318; eBiosience), in 100 μl of 0.05 M carbonate/bicarbonate coating buffer was applied to each well in a 96 well plate. The wells were washed in 1% BSA overnight. 200 μl mAb supernatants (1:1 diluted in TBS-T) or purified mAb were added. Preparations were treated with a horse radish peroxidase conjugate Goat anti-mouse pan IgG-FC Fragment (Cat.# A90-131P; Bethyl Laboratories) in the dark for 1h. Plates were then treated with TMB horse radish peroxidase substrate (Cat.# 50–76.01; Seracare KPL) and assessed at 450 nm in a SpectraMax Plus 384 Microplate Reader (Molecular Devices, San Jose, CA).

### Cellular lysates and protein concentration

Lysates of the cell lines were obtained from 70 to 80% confluent cell cultures. Flasks were washed and cells incubated for 2 hours with intermittent mixing on ice in RIPA lysis buffer (Cat.# 20–188; Millipore-Sigma) supplemented with 50 μM phenylmethylsulphonyl fluoride (PMSF), 1% Triton and the complete mini ethylenediaminetetraacetic acid (EDTA)-free protease inhibitor (Cat.# 11836153001; Roche) and PhosStop (Cat.# 04906845001; Roche). Membranes and cell debris were removed by centrifugation. The protein concentration was assessed using the DC Protein Assay (Cat.# 500–0116; Bio-Rad) according to the manufacturer’s instructions.

### Deglycosylation

Twenty μg of the N-terminal peptide (aa 1–220), expressed in HEK293 cells, were deglycosylated in 80 μl of PBS pH7.4 and 1.6 μl of neuraminidase (Cat.# 1158886001; Millipore-Sigma) and 1.6 μl PNGaseF (Cat.# 9109-GH; R&D Systems), incubated for 1 hour at 37°C and then at 4°C overnight.

### Spot assay

Ten μg cellular lysate or 3 μg peptide were dried on nitrocellulose membranes in the wells of a 6-well plate for at least 1 hour, then treated with Intercept protein free blocking buffer in TBS (Cat.# 927–80001; LI-COR Biosciences) for 1 hour at room temperature. The membranes were then incubated in blocking buffer containing 2.5 μg/mL of the purified mAb for 1 hour at room temperature. The membranes were treated with IR Dye 800-conjugated goat anti-mouse IgG1 secondary antibody (Cat.# 926–32350; LI-COR Biosciences) at a 1:10,000 dilution for 1 hour. The membranes were washed and scanned in a LI-COR Biosciences Fc-Odyssey Scanner (LI-COR Biosciences, Lincoln, NE).

### Western blot

Eighty μg of denatured total cell protein or 3 μg of 80 kDa CD44 recombinant peptide in sample buffer (Cat.# 1610747; Bio-Rad) were separated in a TRIS/glycine/sodium dodecyl sulfate polyacrylamide gel (Cat.# 4561084; Bio-Rad). Dual color molecular weight markers (Cat.# 928–6000; LI-COR) were also separated. Proteins were transferred to nitrocellulose membranes and the blot blocked with Intercept protein free blocking buffer (TBS) overnight. The blocked membranes were then incubated in TBS containing hybridoma supernatant (1:10). Blots were then treated with goat-derived IR Dye 800 anti-mouse antibody (Cat.# 926–32350; LI-COR Biosciences) at a dilution of 1:10,000, and scanned in a LI-COR Biosciences Fc-Odyssey Scanner at 800 nm and 700 nm, respectively.

### Immunofluorescent staining and fluorescent tag

Formaldehyde (3.8%)-fixed cells were stained in TBS (pH 7.6), containing 10 μg per ml of mAb and 1% BSA, then treated with the secondary affinity-purified anti-mouse IgGH+L Alexa Fluor 488 antibody (Cat.# AB-2338840; Jackson Immunoresearch) at a 1:1000 dilution. To visualize expression of the V5-epitope in cells transformed with the expression plasmid pLX304isf12V5, formaldehyde-fixed MB-231 cells were treated with 0.3% TritonX-100. MB-231 cells expressing CD44isf12V5 were imaged through a Nikon TE2000 inverted epifluorescence microscope with a Canon EOS Rebel T3i/EOS 600D camera (Canon, Huntington, NY). Cells stained with mAbs were imaged through a Leica SP8 confocal microscope. Contrast was processed using Fuji software [49].

### Tumorigenesis model

Tumorigenesis experiments were approved by the University of Iowa Institutional Animal Care and Use Committee (IACUC) under Protocol 8121508, in compliance with Public Health Service Policy, Animal Welfare Regulations and the Guide for the Care and Use of Laboratory Animals (Jackson Labs). All efforts were taken to minimize animal suffering. Female NOD.CB17-PRKDCSCID/J mice (Cat.# 001303; Jackson Laboratories) were injected in a posterior mammary fat pad with 1x10^6^ MB-231 cells. To test the effect of the anti-CD44 mAb on tumor formation, affinity-purified anti-CD44 mAb P3D2 was mixed at 5 mg/kg of mouse with the suspension of MB-231 cells, in a final volume of 200 μl, in PBS, pH7.2. As a control, affinity purified IgG1 was mixed with MB-231 cells. Mice were tested in three separate experiments. All mice were euthanized when a palpable tumor was detected in the immediate region of the injection sites of control mice. Mice were dissected, and the mammary fat pads excised. The region of the fat pad with a tumor in control mice and a corresponding region in mAb treated mice were fixed in 4% formaldehyde then transferred to 70% ethanol, paraffin-embedded at the Central Microscopy Research Facility at the University of Iowa, sectioned and stained with hematoxylin and eosin-Y [50].

## Results

### CD44 immunogen

CD44isf1, the isoform representing the complete CD44 open reading frame is composed of an extracellular domain, a transmembrane domain and a cytosolic domain (Fig 1A) [1–5]. The extracellular domain contains a N-terminal conserved region (CED1), which includes the HA binding site, a variable region and a second distal conserved region (CED2) (Fig 1A) (XP 005253288.1 https://www.ncbi.nlm.gov/gene/960). The major factors dictating the size of the CD44 isoforms are the differences in the variable domain (V2-V10) (Fig 1A) [51], located between two conserved domains, which undergoes alternative splicing [40, 41], as well as secondary modifications, most notably glycosylation [1, 4, 5, 52–55]. The molecular weight of the unglycosylated CD44isf1 protein is 81.5 kDa. It has been estimated to have 154 potential glycosylation sites, which predicts the estimated weight of the glycosylated protein to be approximately 120 kDa [4, 5, 37, 40–42, 52] (uniport.org/P16070). The molecular weight of the CD44isf12 protein that lacks the variable region V2-V10 is 39 kDa [37]. The estimated glycosylated molecular weight is approximately 55 kDa (https://www.protpi.ch/. The length of the cytosolic domain has also been demonstrated to vary between isoforms [40, 41, 56].

We previously demonstrated that the anti-CD44 mAb, H4C4 [52, 57] stained all three tested tumorigenic cell lines, the breast cancer cell line MB-231, the melanoma cell line HTB-66, and the glioblastoma cell line U87 [14, 15]. Attempts were made to clone larger CD44 isoforms from all three cell lines using 3Ꞌ RACE and RT-PCR with N-terminal and C-terminal primers, described in S2 Table. The cloned cDNA products lacked a 1147 Bp sequence, which included the variable region V2-V10. Therefore, the 3Ꞌ RACE amplification product represented the isoform CD44isf12. This may have been due to the observation that PCR favors shorter sequences [58]. Alternatively, there may be an abundance of CD44isf12 mRNA. Western blot patterns, described in a later section, suggested the former may be the correct explanation.

### Expression plasmid

The immunization strategy (described below) to generate anti-CD44 mAbs involved injection of both a 15 amino acid peptide of the N-terminal conserved CED2 region, including amino acids 248 to 262, of the proximal conserved domain of CD44isf12 (Fig 1B), and a plasmid that expressed the entire CD44isf12, pLX304isf12V5 (Fig 1C). In the expression plasmid, the CD44isf12 coding sequence is in frame with the DNA sequence of the V5 tag, at the 3Ꞌ-end (CD44isf12V5). The CD44isf12V5 sequence is under the control of the cytomegalovirus (CMV) promoter (Fig 1C) [59, 60].

### Immunization and selection strategies

The strategy for immunization is outlined in Fig 1D. In the immunization regime, the chemically synthesized 15 amino acid peptide (N-GSQEGGANTTSGPIR-C), located in the conserved region CED2 (Fig 1B), was first injected intraperitoneally at one, 14 and 28 days (Fig 1D). Then both the peptide and the expression plasmid pLX304isf12V5 were simultaneously injected at 58 days intraperitoneally and into the tail vein at 70 days (Fig 1D). At 77 days, mice were euthanized (Fig 1D).

### Fusion and hybridoma selection

The spleens of the euthanized mice were isolated and homogenized, the spleenocytes mixed with cells of the immortalized cell line Sp2/0-Ag14 cells [61], fusion induced with 50% PEG and hybridomas isolated by HAT selection (Fig 1E) [62]. Hybridoma clones were then selected for IgG production with anti-IgG1 antibody by fluorescence-activated cell sorting (FACS). Clones were then selected for IgG subtype and for a strong ELISA signal with an 81.5 kDa CD44 peptide, generated in *E*. *coli*, which included the CD44isf1 amino acid sequence. Ten clones satisfied these criteria and four with high growth rates were selected for further analysis.

### Verification of the selected hybridoma mAbs

**Fig 2:**
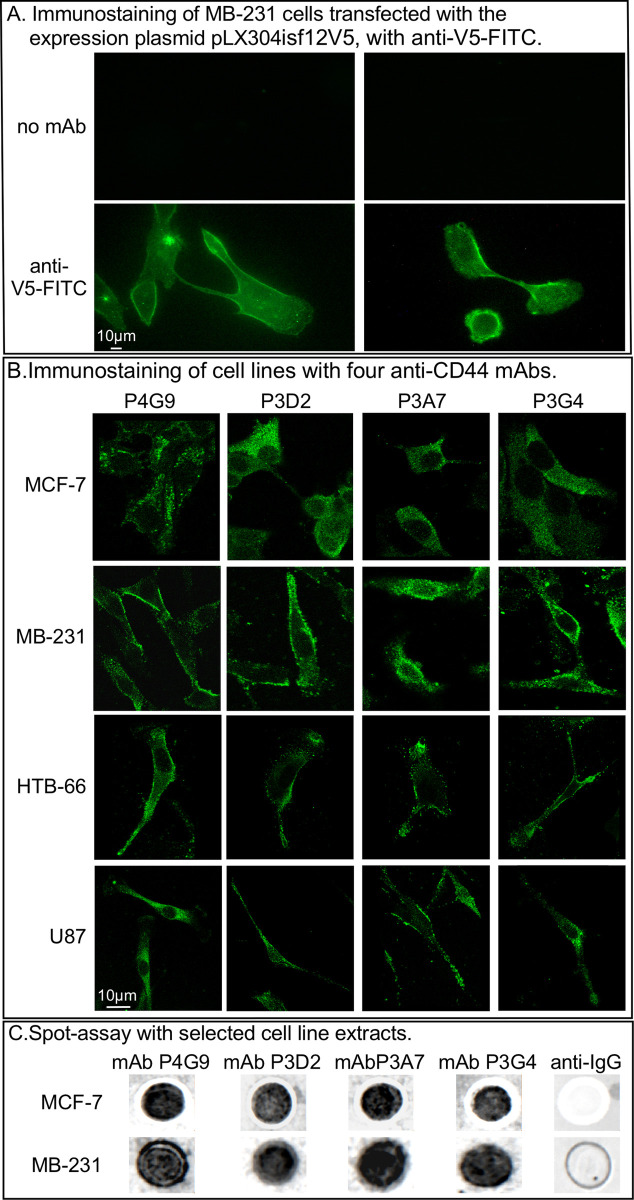
Validation of the expression plasmid, immunocytostaining of cell lines with selected mAbs and spot assay analysis of cell lysates with selected anti-CD44 mAbs. A. Expression of CD44 assessed by immunofluorescence, by the breast cancer cell line transfected with the expression plasmid pLX304isf12V5. Cells were stained with anti-V5-FITC antibody. B. Immunostaining of four cell lines, MCF-7 (breast cancer), MB-231 (breast cancer), HTB-66 (melanoma) and U87 (glioblastoma), with each of the four mAbs, P4G9, P3D2, P3A7, and P3G4. C. Spot assay analysis of cell lysates of the cell lines MCF-7 and MB-231, with the four selected mAbs. Anti-IgG1 was used as a negative control.

Expression of the open reading frame was verified in cells of the breast cancer cell line MB-231. MB-231 cells were transformed with the expression plasmid and immunostained for the V5 tag of CD44isf12, with an anti-V5-FITC antibody. Transformed MB-231 cells stained preferentially in the plasma membrane (Fig 2A), demonstrating that CD44isf12V5 was in fact expressed and localized correctly in human cells transformed with pLX304isf12V5.

Four clones with strong ELISA signals (P4G9, P3A7, P3D2, P3G4) stained fixed cells of four cell lines (MCF-7, nontumorigenic breast; MB-231, tumorigenic breast cancer; HTB-66, tumorigenic melanoma; U87, tumorigenic glioblastoma), demonstrating that the cells of all four cell lines expressed the CD44 target antigen (Fig 2B). For the three cancer cell lines, staining was punctate and localized to the plasma membrane (Fig 2B). The four mAbs were also tested for binding to cell lysates of two lines, nontumorigenic MCF-7 and tumorigenic MB-231, by spot assay analysis. All four mAbs bound to lysates of both cell lines (Fig 2C). Negative controls, probed with anti-IgG, did not bind to the lysates of the cells (Fig 2C).

### Western blot analysis

**Fig 3:**
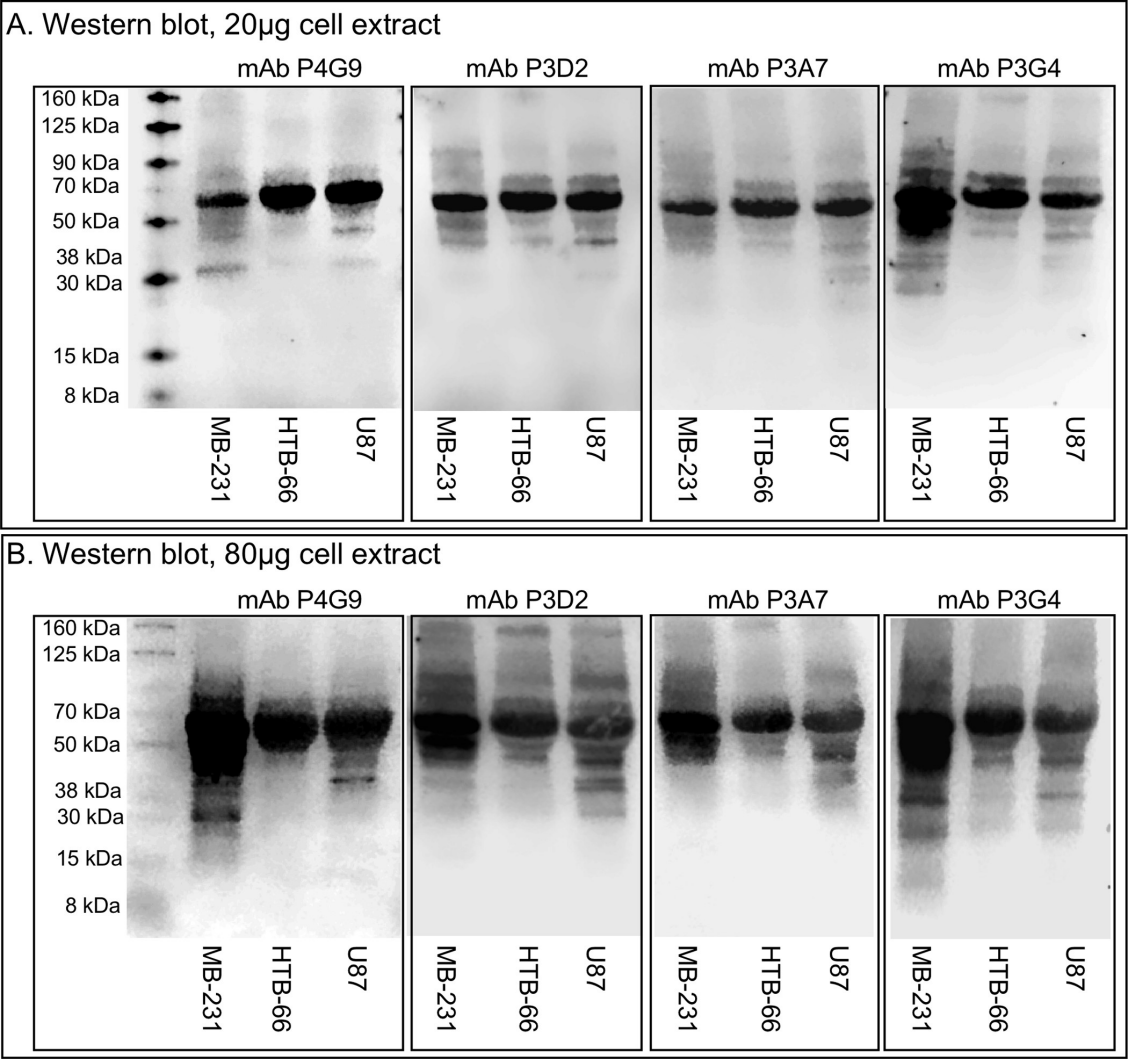
Western blot analyses of the four anti-CD44 mAbs. A. Western blot analysis of lysates of the cell lines MB-231 (breast cancer), HTB-66 (melanoma), and U87 (glioblastoma) loaded with 20 μg total protein and probed with supernatant of the anti-CD44 mAbs P4G9, P3D2, P3A7 and P3G4. B. Western blot similar to that in panel A loaded with 80 μg total protein and probed with supernatant of the four anti-CD44 mAbs.

To assess the variety of isoforms identified by the four selected anti-CD44 mAbs, western blots were performed with lysates of the three tumorigenic cell lines (MB-231, breast cancer; HTB-66, melanoma; U87, glioblastoma). Western blots were loaded with 20 μg (Fig 3A) or 80 μg (Fig 3B), the latter to discriminate minor isoforms. Western blots performed with 20 μg of cell extract of three cell types showed one major band at 60 kDa, except for MB-231 probed with mAb P3G4, which exhibited two major bands at approximately 60 kDa and 50 kDa. By increasing the amount four-fold to 80μg, minor bands could be discriminated and compared in western blots. While there were no obvious differences between P3D2 and P3A7 for each of the three tested strains, there were clear differences between strains (Fig 3B). P4G9 and P3G4 exhibited differences in minor bands, and both exhibited differences when compared to the common pattern of P3D2 and P3A7 (Fig 3B). Together, the results for the western blots loaded with 20 or 80μg of cell extract indicate that P3D2 and P3A7 target the same sequence, while P4G9 and P3G4 target different epitopes of CD44, resulting in three distinguishable antigenic targets, and that all four mAbs discriminate between the three different strains.

### Spot assay with peptides

**Fig 4:**
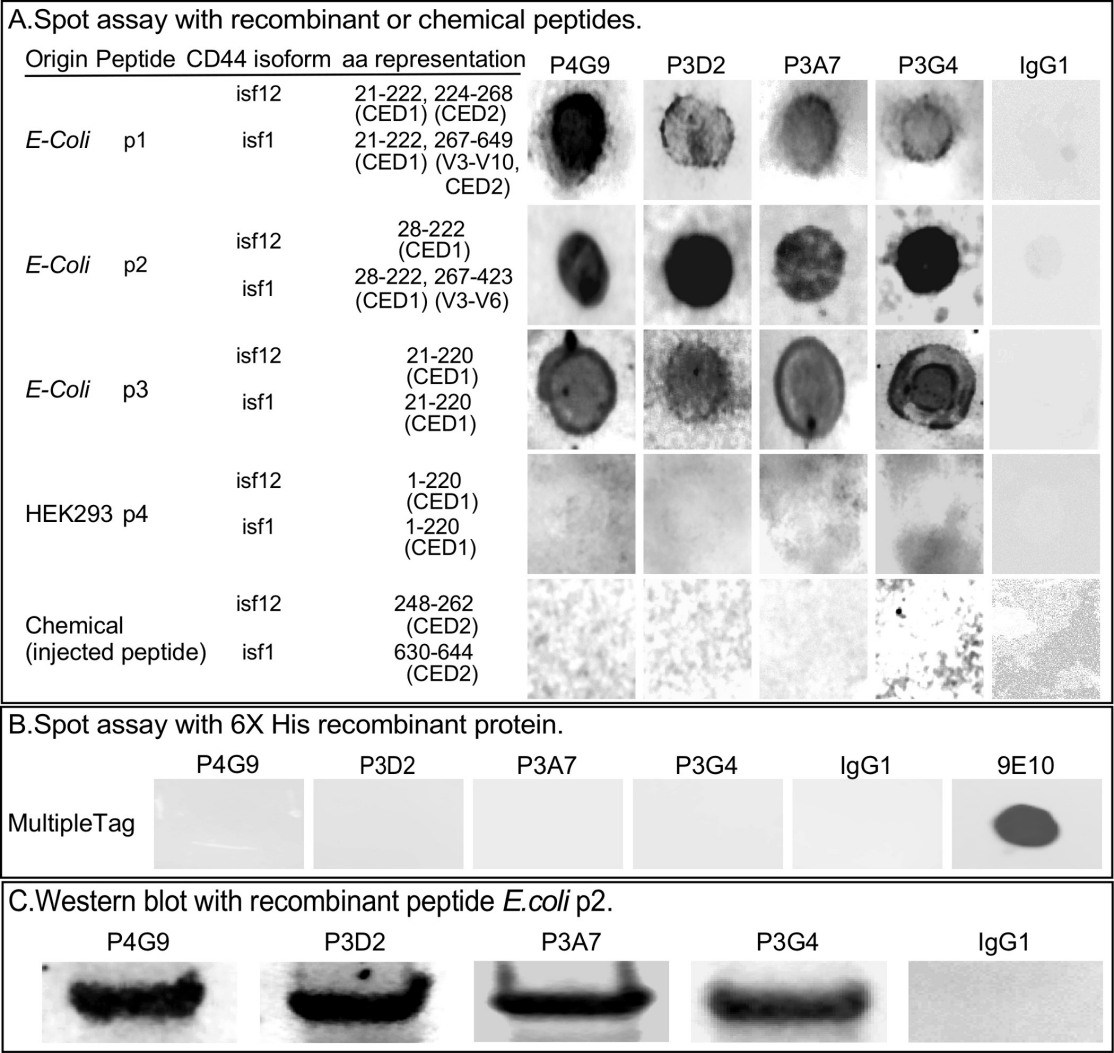
Binding of the four anti-CD44 mAbs to recombinant peptides. A. Spot assay analysis of binding by the four selected anti-CD44 mAbs to three peptides generated in *E*. *coli*, one generated in HEK293 cells and one chemically synthesized. B. Spot assay analysis of binding of the four selected anti-CD44 mAbs to a recombinant multiple tag protein containing 6XHIS. 9E10 is a DSHB mAb against c-MYC in the multiple tag protein. C. Western blot analysis of *E*. *coli* p2 binding to the four generated mAbs. Anti-IgG was used as a negative control.

We next tested binding of the four generated anti-CD44 mAbs to commercially available recombinant CD44 peptides generated in *E*. *coli* and in the human cell line HEK293, and to the chemically synthesized peptide (Fig 1B), which we used in combination with the expression plasmid (Fig 1C) in the immunization protocol (Fig 1D). The first *E*. *coli* recombinant peptide, p1, contained sequences in the conserved domain CED1, (21–222 aas), variable domain V3 to V10, and the conserved domain CED2 (267–649 aas) (Fig 4A). All four anti-CD44 mAbs bound to p1 (Fig 4A). The second *E*. *coli* recombinant peptide, p2, included sequences in CED1 (28–222 aas) and V3-V6 (267–423 aas) (Fig 4A). All four mAbs bound to p2 (Fig 4A). The third *E*. *coli* recombinant peptide, p3, contained only a sequence in CED1 (21–220 aas) (Fig 4A). All four anti-CD44 mAbs bound to p3 (Fig 4A). These results demonstrated that the four selected mAbs recognized target sequences in amino acids 21–220 of CED1. The fourth recombinant peptide, p4, generated in the human cell line HEK293 included aas 1–220 (Fig 4A). Unlike p1, p2 and p3, which were presumably unglycosylated because they were generated in *E*. *coli*, p4 was presumably glycosylated, since it was generated in a mammalian cell line. None of the four anti-CD44 mAbs bound to p4 (Fig 4A). Since the *E*. *coli* recombinant peptides possessed a C-terminal 6XHIS tag, we tested binding of the four anti-CD44 mAbs to a multi-tag peptide containing 6XHIS by spot assay, to exclude the possibility it was the target. None of the four mAbs bound (Fig 4B). A control anti-6XHIS antibody, 9E10, did bind (Fig 4B). Finally, we tested binding to a chemically synthesized 15 amino acid peptide in the CED2 domain, representing aas 630–644 of CD44isf1, which is low in secondary structure and turns, as determined by the Chow-Fasman prediction [63], and antigenic as predicted by the Jameson-Wolf method [64]. None of the four mAbs bound to the chemically synthesized peptide, as one would have predicted based on binding to the previous four peptides (Fig 4A).

### Glycosylation and binding

**Fig 5:**
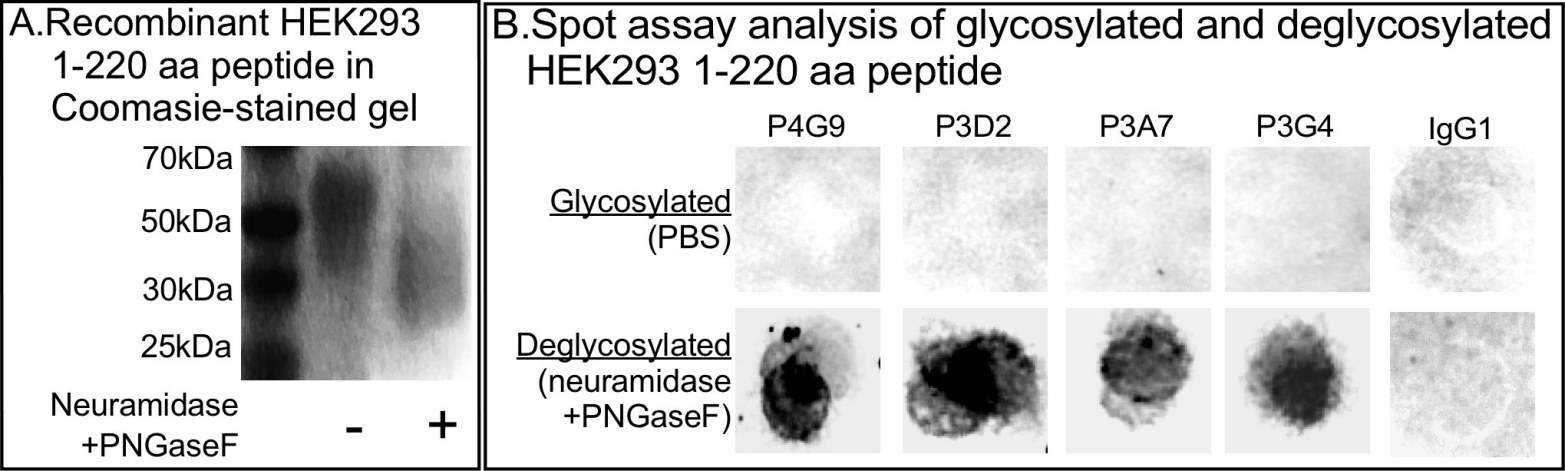
HEK293 recombinant peptide representing the first 220 amino acids of CD44 binds to the four anti-44 mAbs when deglycosylated. A. Treatment of the HEK293 recombinant peptide, that represents amino acids 1–220 of CD44, with neuramidase and PNGaseF causes a decrease in molecular mass from approximately 60 to approximately 40 kDa (-), in the absence, (+) in the presence of neuramidase and PNGaseF. B. A comparison of spot assay binding of the four mAbs, between the glycosylated and deglycosylated HEK293 peptide.

Since the four mAbs were generated against CD44isf12, which lacked the variable region, and bound to the *E*. *coli*-generated peptide p2, which contains only the CED1 sequence, we tentatively concluded that the four mAbs were generated against an amino acid sequences in CED1, which would have been the peptide generated in the expression plasmid pLX304isf12V5, since the co-injected peptide represented a sequence in CED2 (Fig 1B). Western blots of *E*. *coli* p2 probed with the four mAbs supported the results of the spot assay analysis, namely that all four mAbs recognized a sequence in amino acids 28–222 of the CED1 domain (Fig 4C). Since none of the mAbs bound to the HEK293 recombinant peptide, and since the HEK293 recombinant peptide was glycosylated [65, 66], we tested whether deglycosylation of the HEK293 peptide exposed the target aa sequence. We deglycosylated the HEK293 recombinant peptide by treating it with neuraminidase and PNGaseF, which reduced its molecular mass by approximately one-third (Fig 5A), then performed a spot assay comparison of glycosylated and deglycosylated peptides with the four mAbs. While none of the four mAbs bound to the untreated HEK293 peptide, all bound to the Neuraminidase/PNGaseF-treated peptide (Fig 5B). These results supported the conclusion that the four mAbs targeted unglycosylated amino acid sequences in the CD44 conserved domain, CED1.

### Blocking tumorigenesis

**Fig 6:**
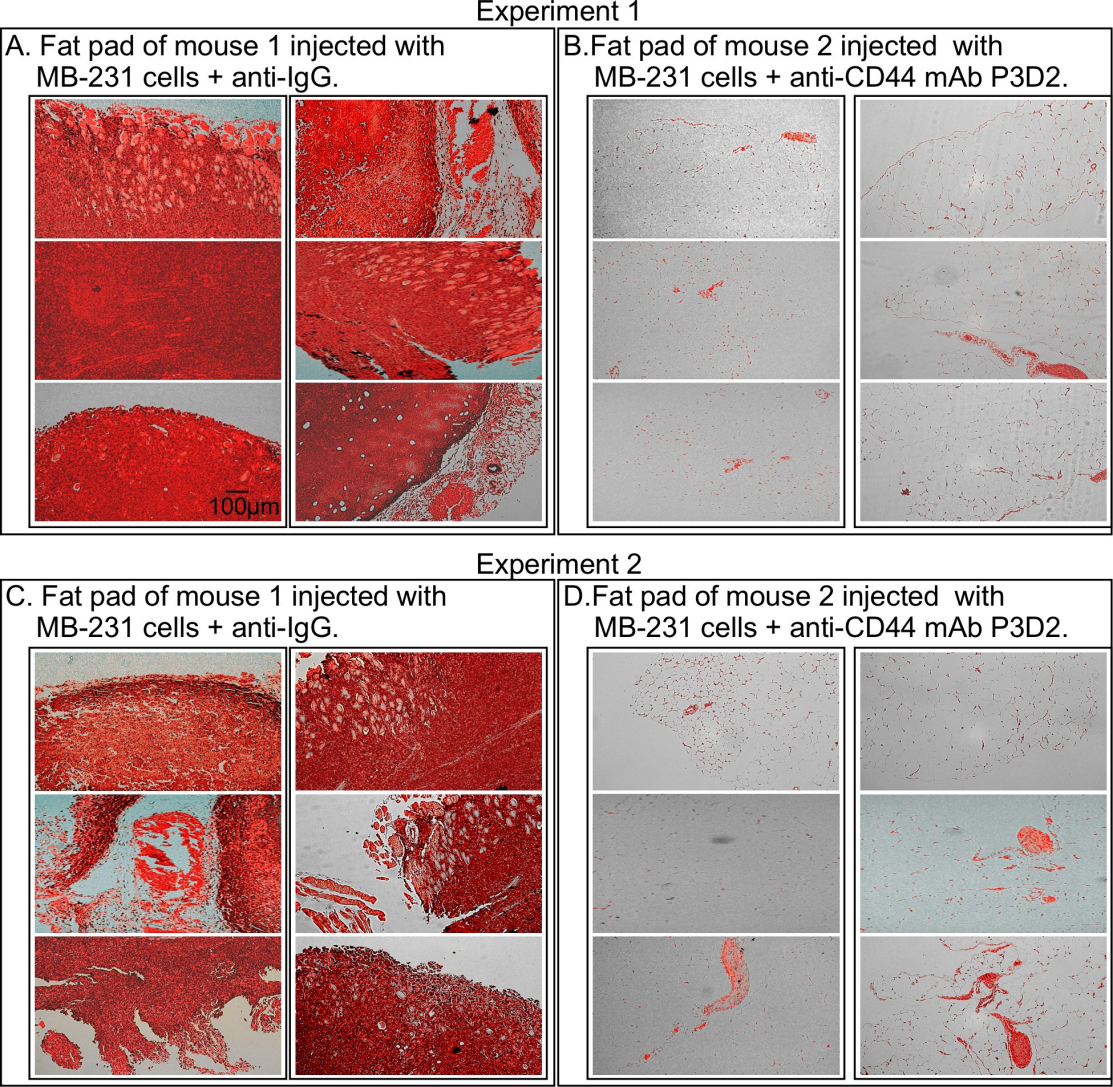
The anti-CD44 mAb P3D2, when injected with cells of the tumorigenic cell line MB-231, blocks tumorigenesis. A mammary fat pad of mice was either injected with tumorigenic plus anti-IgG1 (control) or MB-231 cells plus the anti-CD44 mAb P3D2 (test). When control fat pads contained palpable tumors, the fat pads were removed from euthanized mice, both control and test, and stained with hematoxylin (stains nuclei black) and eosin (stains cytoplasm red). A,C. Representative histological sections of tumors in the mammary fat pads of control mice injected with MB-231 cells and anti-IgG1. B,D. Histological sections of fat pads of test mice injected with MB-231 cells plus the anti-CD44 mAb P3D2.

Since the four generated anti-CD44 mAbs stained fixed cells of all three tested cell lines (Fig 2B) and bound to multiple CD44 isoforms in western blots of cell lysates of each line (Fig 3A), we considered the possibility that the mAbs might recognize native CD44 isoforms *in vivo*. We therefore assessed whether one of the mAbs, P3D2, blocked tumorigenesis by the breast cancer cell line MB-231 in a mouse model. MB-231 cells were mixed with the affinity-purified anti-CD44 mAb P3D2 just prior to injection into mammary fat pads, while in control animals, MB-231 cells were mixed with affinity-purified anti-IgG antibody. For the combined data of three independent experiments, a total of six control animals were injected with MB-231 cells plus anti-IgG (i.e., without mAb P3D2), and a total of six test animals were injected with MB-231 cells and P3D2. Animals were examined twice a week manually for palpable tumors in the mammary fat pads. A palpable large tumor was identified in each of the five control animals after five weeks, at which time both control and test mice were euthanized and the mammary fat pads excised. Of the six test animals, five contained no palpable tumor in the mammary gland and one contained a small palpable tumor. Histological sections of the fat pads, stained with hematoxylin and eosin (H&E), supported this interpretation. Representative histological sections are presented in Fig 6A and 6C for control animals injected with MB-231 cells plus anti-IgG and in Fig 6B and 6D for test animals injected with MB-231 and mAb P3D2. These results demonstrate that the anti-CD44 mAb P3D2, when co-injected with cells of the breast cancer-derived, tumorigenic cell line MB-231 blocked subsequent tumorigenesis.

## Discussion

The large number of CD44 isoforms resulting from alternative splicing and secondary modification is consistent with the variety of cell types and cellular functions [5, 37–42, 51, 67], in which CD44 plays a role [2, 10, 14–18]. CD44 also plays a prominent role in tumor progression and metastasis in a variety of cancers, as reviewed in detail by Chen et al. [13]. Moreover, individual cells express more than one CD44 isoform suggesting that multiple isoforms play a variety of roles in single cells [37, 51, 56]. It would therefore be highly advantageous to generate a highly characterized, diverse panel of anti-CD44 mAbs in order to dissect the roles of the different CD44 isoforms in tumorigenesis and metastasis, and identify specific mAbs that may be useful and selective anti-tumor and anti- metastasis drugs. In addition, it should be noted that different mAbs generated against the same CD44 domain or peptide may vary dramatically in blocking activity or efficacy due to differences in the variable regions of the light and heavy chain, as well as the exact targeted antigenic sequences. Finally, the need for more affinity reagents, most notably monoclonal antibodies and recombinant antibodies, that are well validated and characterized, has engendered initiatives by the NIH and other institutions, such as the Protein Capture Reagents Program (PCRP) of the Common Fund of NIH [68], and the Clinical Proteomic Technologies for Cancer (CPTC) program of the National Cancer Institute of NIH http:proteomics.cancer.gov [69, 70], NeuroMab at UC Davis [71], and the Muscular Dystrophy Association https://www.mda.org [72]. For all of the preceding reasons, we developed a strategy for generating new mAbs against CD44, in which we co-inoculated mice with both a 15 amino acid peptide from the proximal extracellular conserved domain (CED2) of CD44 and an expression plasmid that expressed a protein with the amino acid sequence of the smallest CD44 isoform, isf12, which lacked the extracellular variable region (V2-V10). Ten hybridomas were selected based on IgG subtype, ELISA signals with recombinant CD44 protein and speed of hybridoma growth. The four best of the 10 based on the selection criteria were then characterized.

### mAb variability

The four selected anti-CD44 mAbs each identified a number of isoforms in lysates of the three tested cell lines separated on western blots. Western blot analyses identified three complex patterns, one generated by mAb P4G9, one by both P3D2 and P3A7, and one by P3G4. In addition, each mAb generated different western blot patterns between the three cancer cell lines. These letter results support previous studies demonstrating that different cancer cells express different CD44 isoforms, as do non-cancer cells [16, 23, 24, 27–30, 41, 45]. These, detailed characterizations of isoforms may reveal ones that are specific for cancer cells that can be used as targets for mAbs that selectively block tumorigenesis and metastasis.

### Glycosylation

CD44 isoforms appear to share the 1–222 aas that compose the conserved N-terminal extracellular domain (CED1). Sequences in the 21 to 220 domain proved to be the antigenic targets for all four cloned mAbs. A search for N- and O-glycosylation for CD44isf12 at http://www.cbs.dtu.dk revealed 24 potential O-glycosylation sites along CD44, including at least 5 in the CED1 domain. These potential sites may explain why the four generated mAbs bound to the unglycosylated *E*. *coli* recombinant peptide p3 (aas 21–220), but not the glycosylated HEK293 recombinant protein (aas 1–220). Deglycosylation of the HEK293 recombinant peptide allowed the four generated anti-CD44 mAbs to bind to the peptide, as demonstrated by spot assay analysis. It should be noted that glycosylation of the recombinant peptide when it was generated in HEK293 cells may be different from that of native CD44 isoforms generated in the three cancer cell lines [65]. Western blots of cell line lysates indicated that each of the four mAbs bound to a number of different native CD44 isoforms, presumably by binding to the same unglycosylated amino acid sequences of CD44, suggesting that the CED1 sequence glycosylated in the recombinant HEK293 peptide was not glycosylated in the native CD44 isoforms identified by western blot analysis of lysates of all three cancer cell lines. Interestingly, Matsuki et al. (2003) generated a monoclonal antibody, 268-1F5, that bound to a sequence in the proximal conserved CED2 region of CD44 [73], that recognized CD44 only after deglycosylation. In western blot analysis of cell line lysates including that of MB-231, the 268-1F5 mAb produced only a weak signal with an 85kDa isoform, it’s major target [73]. In contrast, the major target of the four anti-CD44 mAbs we have generated is approximately 65 kDa and exhibits a strong signal in MB-231 cell lysates probed with the 4 generated mAbs. These results suggest that other anti-CD44 mAbs generated against different conserved regions using unglycosylated recombinant proteins may be less effective due to glycosylation of the target domains in the mature protein.

### P3D2 inhibits tumorigenesis

Here, we have tested whether one of the anti-CD44 mAbs we generated, P3D2, was anti-tumorigenic. To test for anti-tumorigenic activity, we co-injected the mAb P3D2 with cells of the tumorigenic cell line MB-231 [74–76], into the mammary fat pads of mice. P3D2 was highly effective in blocking tumorigenesis by MB-231 cells.

### Concluding remarks

The CD44 isoforms not only play major roles in a host of cellular functions ranging from embryogenesis to the cell physiology of adults in a variety of tissues, but also in cancer tumorigenesis [77]. CD44 knockdown experiments and anti-CD44 antibodies have been shown to affect a number of cancer cell-related behaviors *in vitro* [14, 78, 79]. Several previous studies demonstrated that mAbs against CD44 blocked tumorigenesis in mouse models [14, 32, 80, 81]. In the study by Verel et al. (2002), the target epitope binding region of the mAbs were in the variable region of CD44 between aa 36–370 [80]. None of the studies assessed the role of glycosylation, as we have performed here. We have shown that the mAbs we have generated identify unglycosylated sequences in the N-terminal conserved region of native CD44 isoforms. The four characterized mAbs bind to a variety of CD44 isoforms in cancer cell lysates, indicating that the targeted amino acids region of these isoforms, which is in the N-terminal conserved region, is unglycosylated in the native state. Here, we have tested one of the four mAbs for its capacity to inhibit tumorigenesis by breast cancer cells in the mammary fat pad of mice. The antibody was highly effective, suggesting that mAbs targeting the unglycosylated conserved N-terminal region of CD44 may prove clinically relevant. Interestingly, CD44 generated in HEK293 cells, which were cloned from human embryonic kidney, was glycosylated in the mAb target region of CD44, indicating that cancer cells leave this region unglycosylated and possibly, this provides a unique cancer cell target. This possibility is now under investigation.

## Supporting information

S1 Table Anti-CD44 monoclonal Abs availability. (DOCX)

S2 Table Primer sequences and location of binding. (DOCX)

S1 Raw images (PDF)

## References

[1] Jacobson K, O’Dell D, Holifield B, Murphy TL, August JT. Redistribution of a major cell surface glycoprotein during cell movement. J Cell Biol. 1984;99(5):1613–23. Epub 1984/11/01. 10.1083/jcb.99.5.1613 6386823 PMC2113370

[2] Wayner EA, Carter WG, Piotrowicz RS, Kunicki TJ. The function of multiple extracellular matrix receptors in mediating cell adhesion to extracellular matrix: preparation of monoclonal antibodies to the fibronectin receptor that specifically inhibit cell adhesion to fibronectin and react with platelet glycoproteins Ic-IIa. J Cell Biol. 1988;107(5):1881–91. Epub 1988/11/01. 10.1083/jcb.107.5.1881 2846588 PMC2115330

[3] Jalkanen ST, Bargatze RF, Herron LR, Butcher EC. A lymphoid cell surface glycoprotein involved in endothelial cell recognition and lymphocyte homing in man. Eur J Immunol. 1986;16(10):1195–202. Epub 1986/10/01. 10.1002/eji.1830161003 . 2429846

[4] Goldstein LA, Zhou DF, Picker LJ, Minty CN, Bargatze RF, Ding JF, et al. A human lymphocyte homing receptor, the hermes antigen, is related to cartilage proteoglycan core and link proteins. Cell. 1989;56(6):1063–72. Epub 1989/03/24. 10.1016/0092-8674(89)90639-9 . 2466576

[5] Zhou DF, Ding JF, Picker LJ, Bargatze RF, Butcher EC, Goeddel DV. Molecular cloning and expression of Pgp-1. The mouse homolog of the human H-CAM (Hermes) lymphocyte homing receptor. J Immunol. 1989;143(10):3390–5. Epub 1989/11/15. . 2681416

[6] Aruffo A, Stamenkovic I, Melnick M, Underhill CB, Seed B. CD44 is the principal cell surface receptor for hyaluronate. Cell. 1990;61(7):1303–13. Epub 1990/06/29. 10.1016/0092-8674(90)90694-a . 1694723

[7] Miyake K, Underhill CB, Lesley J, Kincade PW. Hyaluronate can function as a cell adhesion molecule and CD44 participates in hyaluronate recognition. J Exp Med. 1990;172(1):69–75. Epub 1990/07/01. 10.1084/jem.172.1.69 2193100 PMC2188161

[8] Culty M, Nguyen HA, Underhill CB. The hyaluronan receptor (CD44) participates in the uptake and degradation of hyaluronan. J Cell Biol. 1992;116(4):1055–62. Epub 1992/02/01. 10.1083/jcb.116.4.1055 1370836 PMC2289343

[9] Underhill C. CD44: the hyaluronan receptor. J Cell Sci. 1992;103 (Pt 2):293–8. Epub 1992/10/01. . 1282514 10.1242/jcs.103.2.293

[10] Weber GF, Ashkar S, Glimcher MJ, Cantor H. Receptor-ligand interaction between CD44 and osteopontin (Eta-1). Science. 1996;271(5248):509–12. Epub 1996/01/26. 10.1126/science.271.5248.509 . 8560266

[11] Ahrens T, Sleeman JP, Schempp CM, Howells N, Hofmann M, Ponta H, et al. Soluble CD44 inhibits melanoma tumor growth by blocking cell surface CD44 binding to hyaluronic acid. Oncogene. 2001;20(26):3399–408. Epub 2001/06/26. 10.1038/sj.onc.1204435 . 11423990

[12] Hua Q, Knudson CB, Knudson W. Internalization of hyaluronan by chondrocytes occurs via receptor-mediated endocytosis. J Cell Sci. 1993;106 (Pt 1):365–75. Epub 1993/09/01. . 7505784 10.1242/jcs.106.1.365

[13] Chen C, Zhao S, Karnad A, Freeman JW. The biology and role of CD44 in cancer progression: therapeutic implications. J Hematol Oncol. 2018;11(1):64. Epub 2018/05/12. 10.1186/s13045-018-0605-5 29747682 PMC5946470

[14] Wessels D, Lusche DF, Voss E, Kuhl S, Buchele EC, Klemme MR, et al. Melanoma cells undergo aggressive coalescence in a 3D Matrigel model that is repressed by anti-CD44. PLoS One. 2017;12(3):e0173400. Epub 2017/03/07. 10.1371/journal.pone.0173400 28264026 PMC5338862

[15] Lusche DF, Klemme MR, Soll BA, Reis RJ, Forrest CC, Nop TS, et al. Integrin alpha-3 ss-1’s central role in breast cancer, melanoma and glioblastoma cell aggregation revealed by antibodies with blocking activity. MAbs. 2019. Epub 2019/02/28. 10.1080/19420862.2019.1583987 . 30810437 PMC6601557

[16] Papatheodorou I, Moreno P, Manning J, Fuentes AM, George N, Fexova S, et al. Expression Atlas update: from tissues to single cells. Nucleic Acids Res. 2020;48(D1):D77–D83. Epub 2019/10/31. 10.1093/nar/gkz947 31665515 PMC7145605

[17] Flanagan BF, Dalchau R, Allen AK, Daar AS, Fabre JW. Chemical composition and tissue distribution of the human CDw44 glycoprotein. Immunology. 1989;67(2):167–75. Epub 1989/06/01. 2666306 PMC1385252

[18] Wang C, Tammi M, Tammi R. Distribution of hyaluronan and its CD44 receptor in the epithelia of human skin appendages. Histochemistry. 1992;98(2):105–12. Epub 1992/09/01. 10.1007/BF00717001 . 1429018

[19] Al-Hajj M, Wicha MS, Benito-Hernandez A, Morrison SJ, Clarke MF. Prospective identification of tumorigenic breast cancer cells. Proc Natl Acad Sci U S A. 2003;100(7):3983–8. Epub 2003/03/12. 10.1073/pnas.0530291100 12629218 PMC153034

[20] Pietras A, Katz AM, Ekstrom EJ, Wee B, Halliday JJ, Pitter KL, et al. Osteopontin-CD44 signaling in the glioma perivascular niche enhances cancer stem cell phenotypes and promotes aggressive tumor growth. Cell Stem Cell. 2014;14(3):357–69. Epub 2014/03/13. 10.1016/j.stem.2014.01.005 24607407 PMC3999042

[21] Kong Y, Lyu N, Wu J, Tang H, Xie X, Yang L, et al. Breast cancer stem cell markers CD44 and ALDH1A1 in serum: distribution and prognostic value in patients with primary breast cancer. J Cancer. 2018;9(20):3728–35. Epub 2018/11/09. 10.7150/jca.28032 30405844 PMC6215997

[22] Jin L, Hope KJ, Zhai Q, Smadja-Joffe F, Dick JE. Targeting of CD44 eradicates human acute myeloid leukemic stem cells. Nat Med. 2006;12(10):1167–74. Epub 2006/09/26. 10.1038/nm1483 . 16998484

[23] Gunthert U, Hofmann M, Rudy W, Reber S, Zoller M, Haussmann I, et al. A new variant of glycoprotein CD44 confers metastatic potential to rat carcinoma cells. Cell. 1991;65(1):13–24. Epub 1991/04/05. 10.1016/0092-8674(91)90403-l . 1707342

[24] Hofmann M, Rudy W, Zoller M, Tolg C, Ponta H, Herrlich P, et al. CD44 splice variants confer metastatic behavior in rats: homologous sequences are expressed in human tumor cell lines. Cancer Res. 1991;51(19):5292–7. Epub 1991/10/01. . 1717145

[25] Stamenkovic I, Amiot M, Pesando JM, Seed B. A lymphocyte molecule implicated in lymph node homing is a member of the cartilage link protein family. Cell. 1989;56(6):1057–62. Epub 1989/03/24. 10.1016/0092-8674(89)90638-7 . 2466575

[26] Ishii S, Ford R, Thomas P, Nachman A, Steele G Jr., Jessup JM. CD44 participates in the adhesion of human colorectal carcinoma cells to laminin and type IV collagen. Surg Oncol. 1993;2(4):255–64. Epub 1993/08/01. 10.1016/0960-7404(93)90015-q . 7504563

[27] Iida N, Bourguignon LY. New CD44 splice variants associated with human breast cancers. J Cell Physiol. 1995;162(1):127–33. Epub 1995/01/01. 10.1002/jcp.1041620115 . 7529235

[28] Kainz C, Kohlberger P, Sliutz G, Tempfer C, Heinzl H, Reinthaller A, et al. Splice variants of CD44 in human cervical cancer stage IB to IIB. Gynecol Oncol. 1995;57(3):383–7. Epub 1995/06/01. 10.1006/gyno.1995.1159 . 7539775

[29] Kaufmann M, Heider KH, Sinn HP, von Minckwitz G, Ponta H, Herrlich P. CD44 variant exon epitopes in primary breast cancer and length of survival. Lancet. 1995;345(8950):615–9. Epub 1995/03/11. 10.1016/s0140-6736(95)90521-9 . 7534855

[30] Yamaguchi A, Saito M, Gio T, Iida A, Takeuchi K, Hirose K, et al. Expression of CD44 variant exons 8–10 in gastric cancer. Jpn J Cancer Res. 1995;86(12):1166–71. Epub 1995/12/01. 10.1111/j.1349-7006.1995.tb03310.x 8636005 PMC5920665

[31] Fonseca I, Pereira T, Rosa-Santos J, Soares J. Expression of CD44 isoforms in squamous cell carcinoma of the border of the tongue: A correlation with histological grade, pattern of stromal invasion, and cell differentiation. J Surg Oncol. 2001;76(2):115–20. Epub 2001/02/27. 10.1002/1096-9098(200102)76:2<115::aid-jso1021>3.0.co;2-9 . 11223837

[32] Guo Y, Ma J, Wang J, Che X, Narula J, Bigby M, et al. Inhibition of human melanoma growth and metastasis in vivo by anti-CD44 monoclonal antibody. Cancer Res. 1994;54(6):1561–5. Epub 1994/03/15. . 7511044

[33] Seiter S, Arch R, Reber S, Komitowski D, Hofmann M, Ponta H, et al. Prevention of tumor metastasis formation by anti-variant CD44. J Exp Med. 1993;177(2):443–55. Epub 1993/02/01. 10.1084/jem.177.2.443 8426113 PMC2190906

[34] Godar S, Ince TA, Bell GW, Feldser D, Donaher JL, Bergh J, et al. Growth-inhibitory and tumor- suppressive functions of p53 depend on its repression of CD44 expression. Cell. 2008;134(1):62–73. Epub 2008/07/11. 10.1016/j.cell.2008.06.006 18614011 PMC3222460

[35] Van Phuc P, Nhan PL, Nhung TH, Tam NT, Hoang NM, Tue VG, et al. Downregulation of CD44 reduces doxorubicin resistance of CD44CD24 breast cancer cells. Onco Targets Ther. 2011;4:71–8. Epub 2011/07/28. 10.2147/OTT.S21431 21792314 PMC3143907

[36] Biddle A, Gammon L, Fazil B, Mackenzie IC. CD44 staining of cancer stem-like cells is influenced by down-regulation of CD44 variant isoforms and up-regulation of the standard CD44 isoform in the population of cells that have undergone epithelial-to-mesenchymal transition. PLoS One. 2013;8(2):e57314. Epub 2013/02/26. 10.1371/journal.pone.0057314 23437366 PMC3577706

[37] St John T, Gallatin WM, Idzerda RL. CD44 expression results in the synthesis of unusual RNAs and multiple protein species containing core polypeptide sequences. Reg Immunol. 1989;2(5):300–10. Epub 1989/09/01. . 2485680

[38] Nottenburg C, Rees G, St John T. Isolation of mouse CD44 cDNA: structural features are distinct from the primate cDNA. Proc Natl Acad Sci U S A. 1989;86(21):8521–5. Epub 1989/11/01. 10.1073/pnas.86.21.8521 2682651 PMC298314

[39] Idzerda RL, Carter WG, Nottenburg C, Wayner EA, Gallatin WM, St John T. Isolation and DNA sequence of a cDNA clone encoding a lymphocyte adhesion receptor for high endothelium. Proc Natl Acad Sci U S A. 1989;86(12):4659–63. Epub 1989/06/01. 10.1073/pnas.86.12.4659 2471974 PMC287330

[40] Jackson DG, Buckley J, Bell JI. Multiple variants of the human lymphocyte homing receptor CD44 generated by insertions at a single site in the extracellular domain. J Biol Chem. 1992;267(7):4732–9. Epub 1992/03/05. . 1537855

[41] Cooper DL, Dougherty G, Harn HJ, Jackson S, Baptist EW, Byers J, et al. The complex CD44 transcriptional unit; alternative splicing of three internal exons generates the epithelial form of CD44. Biochem Biophys Res Commun. 1992;182(2):569–78. Epub 1992/01/31. 10.1016/0006-291x(92)91770-q . 1734871

[42] Azevedo R, Gaiteiro C, Peixoto A, Relvas-Santos M, Lima L, Santos LL, et al. CD44 glycoprotein in cancer: a molecular conundrum hampering clinical applications. Clin Proteomics. 2018;15:22. Epub 2018/07/10. 10.1186/s12014-018-9198-9 29983670 PMC6020424

[43] Wang Z, Wu Y, Wang H, Zhang Y, Mei L, Fang X, et al. Interplay of mevalonate and Hippo pathways regulates RHAMM transcription via YAP to modulate breast cancer cell motility. Proc Natl Acad Sci U S A. 2014;111(1):E89–98. Epub 2013/12/25. 10.1073/pnas.1319190110 24367099 PMC3890879

[44] Xu H, Tian Y, Yuan X, Liu Y, Wu H, Liu Q, et al. Enrichment of CD44 in basal-type breast cancer correlates with EMT, cancer stem cell gene profile, and prognosis. Onco Targets Ther. 2016;9:431–44. Epub 2016/02/09. 10.2147/OTT.S97192 26855592 PMC4727509

[45] Brown RL, Reinke LM, Damerow MS, Perez D, Chodosh LA, Yang J, et al. CD44 splice isoform switching in human and mouse epithelium is essential for epithelial-mesenchymal transition and breast cancer progression. J Clin Invest. 2011;121(3):1064–74. Epub 2011/03/12. 10.1172/JCI44540 21393860 PMC3049398

[46] Lusche DF, Buchele EC, Russell KB, Soll BA, Vitolo MI, Klemme MR, et al. Overexpressing TPTE2 (TPIP), a homolog of the human tumor suppressor gene PTEN, rescues the abnormal phenotype of the PTEN(-/-) mutant. Oncotarget. 2018;9(30):21100–21. Epub 2018/05/17. 10.18632/oncotarget.24941 29765523 PMC5940379

[47] Sanchez P, Daniels KJ, Park YN, Soll DR. Generating a battery of monoclonal antibodies against native green fluorescent protein for immunostaining, FACS, IP, and ChIP using a unique adjuvant. Monoclon Antib Immunodiagn Immunother. 2014;33(2):80–8. Epub 2014/04/22. 10.1089/mab.2013.0089 24746148 PMC3998673

[48] Park YN, Glover RA, Daniels KJ, Soll DR. Generation and Validation of Monoclonal Antibodies Against the Maltose Binding Protein. Monoclon Antib Immunodiagn Immunother. 2016;35(2):104–8. Epub 2016/03/17. 10.1089/mab.2015.0072 26982821 PMC4845686

[49] Schindelin J, Arganda-Carreras I, Frise E, Kaynig V, Longair M, Pietzsch T, et al. Fiji: an open-source platform for biological-image analysis. Nat Methods. 2012;9(7):676–82. Epub 2012/06/30. 10.1038/nmeth.2019 22743772 PMC3855844

[50] Harrris HF. On the rapid conversion of haematoxylin into haematein in staining reactions. JApplMicrosc. 1900;3:777–80.

[51] Tolg C, Hofmann M, Herrlich P, Ponta H. Splicing choice from ten variant exons establishes CD44 variability. Nucleic Acids Res. 1993;21(5):1225–9. Epub 1993/03/11. 10.1093/nar/21.5.1225 8464707 PMC309286

[52] Hughes EN, August JT. Characterization of plasma membrane proteins identified by monoclonal antibodies. J Biol Chem. 1981;256(2):664–71. Epub 1981/01/25. . 7451466

[53] Stamenkovic I, Aruffo A, Amiot M, Seed B. The hematopoietic and epithelial forms of CD44 are distinct polypeptides with different adhesion potentials for hyaluronate-bearing cells. EMBO J. 1991;10(2):343–8. Epub 1991/02/01. 1991450 10.1002/j.1460-2075.1991.tb07955.x PMC452652

[54] Picker LJ, Nakache M, Butcher EC. Monoclonal antibodies to human lymphocyte homing receptors define a novel class of adhesion molecules on diverse cell types. J Cell Biol. 1989;109(2):927–37. Epub 1989/08/01. 10.1083/jcb.109.2.927 2474557 PMC2115731

[55] Picker LJ, De los Toyos J, Telen MJ, Haynes BF, Butcher EC. Monoclonal antibodies against the CD44 [In(Lu)-related p80], and Pgp-1 antigens in man recognize the Hermes class of lymphocyte homing receptors. J Immunol. 1989;142(6):2046–51. Epub 1989/03/15. . 2646376

[56] Screaton GR, Bell MV, Jackson DG, Cornelis FB, Gerth U, Bell JI. Genomic structure of DNA encoding the lymphocyte homing receptor CD44 reveals at least 12 alternatively spliced exons. Proc Natl Acad Sci U S A. 1992;89(24):12160–4. Epub 1992/12/15. 10.1073/pnas.89.24.12160 1465456 PMC50718

[57] Belitsos PC, Hildreth JE, August JT. Homotypic cell aggregation induced by anti-CD44(Pgp-1) monoclonal antibodies and related to CD44(Pgp-1) expression. J Immunol. 1990;144(5):1661–70. Epub 1990/03/01. . 1689752

[58] Kalle E, Kubista M, Rensing C. Multi-template polymerase chain reaction. Biomol Detect Quantif. 2014;2:11–29. Epub 2014/12/04. 10.1016/j.bdq.2014.11.002 27896140 PMC5121205

[59] Thomsen DR, Stenberg RM, Goins WF, Stinski MF. Promoter-regulatory region of the major immediate early gene of human cytomegalovirus. Proc Natl Acad Sci U S A. 1984;81(3):659–63. Epub 1984/02/01. 10.1073/pnas.81.3.659 6322160 PMC344894

[60] Foecking MK, Hofstetter H. Powerful and versatile enhancer-promoter unit for mammalian expression vectors. Gene. 1986;45(1):101–5. Epub 1986/01/01. 10.1016/0378-1119(86)90137-x . 3023199

[61] Shulman M, Wilde CD, Kohler G. A better cell line for making hybridomas secreting specific antibodies. Nature. 1978;276(5685):269–70. Epub 1978/11/16. 10.1038/276269a0 . 714156

[62] Holliday R, Ho T. Evidence for gene silencing by endogenous DNA methylation. Proc Natl Acad Sci U S A. 1998;95(15):8727–32. Epub 1998/07/22. 10.1073/pnas.95.15.8727 9671746 PMC21144

[63] Chou PY, Fasman GD. Prediction of the secondary structure of proteins from their amino acid sequence. Adv Enzymol Relat Areas Mol Biol. 1978;47:45–148. Epub 1978/01/01. 10.1002/9780470122921.ch2 . 364941

[64] Jameson BA, Wolf H. The antigenic index: a novel algorithm for predicting antigenic determinants. Comput Appl Biosci. 1988;4(1):181–6. Epub 1988/03/01. 10.1093/bioinformatics/4.1.181 . 2454713

[65] Croset A, Delafosse L, Gaudry JP, Arod C, Glez L, Losberger C, et al. Differences in the glycosylation of recombinant proteins expressed in HEK and CHO cells. J Biotechnol. 2012;161(3):336–48. Epub 2012/07/21. 10.1016/j.jbiotec.2012.06.038 . 22814405

[66] Goh JB, Ng SK. Impact of host cell line choice on glycan profile. Crit Rev Biotechnol. 2018;38(6):851–67. Epub 2017/12/22. 10.1080/07388551.2017.1416577 . 29262720

[67] Olsson E, Honeth G, Bendahl PO, Saal LH, Gruvberger-Saal S, Ringner M, et al. CD44 isoforms are heterogeneously expressed in breast cancer and correlate with tumor subtypes and cancer stem cell markers. BMC Cancer. 2011;11:418. Epub 2011/10/01. 10.1186/1471-2407-11-418 21957977 PMC3196967

[68] Blackshaw S, Venkataraman A, Irizarry J, Yang K, Anderson S, Campbell E, et al. The NIH Protein Capture Reagents Program (PCRP): a standardized protein affinity reagent toolbox. Nat Methods. 2016;13(10):805–6. Epub 2016/09/30. 10.1038/nmeth.4013 27684578 PMC5886368

[69] Tao F. 1st NCI annual meeting on Clinical Proteomic Technologies for Cancer. Expert Rev Proteomics. 2008;5(1):17–20. Epub 2008/02/20. 10.1586/14789450.5.1.17 . 18282119

[70] Schoenherr RM, Huang D, Voytovich UJ, Ivey RG, Kennedy JJ, Saul RG, et al. A dataset describing a suite of novel antibody reagents for the RAS signaling network. Sci Data. 2019;6(1):160. Epub 2019/08/31. 10.1038/s41597-019-0166-7 31467290 PMC6715692

[71] Gong B, Murray KD, Trimmer JS. Developing high-quality mouse monoclonal antibodies for neuroscience research—approaches, perspectives and opportunities. N Biotechnol. 2016;33(5 Pt A):551–64. Epub 2015/12/09. 10.1016/j.nbt.2015.11.007 26644354 PMC4884554

[72] Morris G, Man N, Sewry CA. Monitoring duchenne muscular dystrophy gene therapy with epitope-specific monoclonal antibodies. Methods Mol Biol. 2011;709:39–61. Epub 2011/01/05. 10.1007/978-1-61737-982-6_3 . 21194020

[73] Matsuki H, Yonezawa K, Obata K, Iwata K, Nakamura H, Okada Y, et al. Monoclonal antibodies with defined recognition sequences in the stem region of CD44: detection of differential glycosylation of CD44 between tumor and stromal cells in tissue. Cancer Res. 2003;63(23):8278–83. Epub 2003/12/18. . 14678986

[74] Cailleau R, Young R, Olive M, Reeves WJ Jr. Breast tumor cell lines from pleural effusions. J Natl Cancer Inst. 1974;53(3):661–74. Epub 1974/09/01. 10.1093/jnci/53.3.661 4412247 PMC7364228

[75] Mullen P, Ritchie A, Langdon SP, Miller WR. Effect of Matrigel on the tumorigenicity of human breast and ovarian carcinoma cell lines. Int J Cancer. 1996;67(6):816–20. Epub 1996/09/17. 10.1002/(SICI)1097-0215(19960917)67:6<816::AID-IJC10>3.0.CO;2-# . 8824553

[76] Wessels DJ, Pradhan N, Park YN, Klepitsch MA, Lusche DF, Daniels KJ, et al. Reciprocal signaling and direct physical interactions between fibroblasts and breast cancer cells in a 3D environment. PLoS One. 2019;14(6):e0218854. Epub 2019/06/25. 10.1371/journal.pone.0218854 31233557 PMC6590889

[77] Xu H, Niu M, Yuan X, Wu K, Liu A. CD44 as a tumor biomarker and therapeutic target. Exp Hematol Oncol. 2020;9(1):36. Epub 2020/12/12. 10.1186/s40164-020-00192-0 33303029 PMC7727191

[78] Wang Z, von Au A, Schnolzer M, Hackert T, Zoller M. CD44v6-competent tumor exosomes promote motility, invasion and cancer-initiating cell marker expression in pancreatic and colorectal cancer cells. Oncotarget. 2016;7(34):55409–36. Epub 2016/07/16. 10.18632/oncotarget.10580 27419629 PMC5342426

[79] Li L, Hao X, Qin J, Tang W, He F, Smith A, et al. Antibody against CD44s inhibits pancreatic tumor initiation and postradiation recurrence in mice. Gastroenterology. 2014;146(4):1108–18. Epub 2014/01/09. 10.1053/j.gastro.2013.12.035 24397969 PMC3982149

[80] Verel I, Heider KH, Siegmund M, Ostermann E, Patzelt E, Sproll M, et al. Tumor targeting properties of monoclonal antibodies with different affinity for target antigen CD44V6 in nude mice bearing head-and-neck cancer xenografts. Int J Cancer. 2002;99(3):396–402. Epub 2002/05/07. 10.1002/ijc.10369 . 11992408

[81] Marangoni E, Lecomte N, Durand L, de Pinieux G, Decaudin D, Chomienne C, et al. CD44 targeting reduces tumour growth and prevents post-chemotherapy relapse of human breast cancers xenografts. Br J Cancer. 2009;100(6):918–22. Epub 2009/02/26. 10.1038/sj.bjc.6604953 19240712 PMC2661796

