# Supplementary material for: New monoclonal antibodies that recognize an unglycosylated, conserved, extracellular region of CD44 in vitro and in vivo, and can block tumorigenesis
Source: PLoS One. 2021 Apr 23;16(4):e0250175. doi: 10.1371/journal.pone.0250175 (PMC8064539; doi:10.1371/journal.pone.0250175)
Supplement: S1 Table — (DOCX) [file pone.0250175.s001.docx]

S1 Table: anti-CD44 monoclonal Abs availability.

Compilation of 140 available mAbs taken from the validated antibody database (VAD; Mater Methods 2016;6:1492). *Ig, Immunoglobulin; aa, amino acid; **v, variant.

| Clone | Company | Host | Isoform | Immunogen |  |
| --- | --- | --- | --- | --- | --- |
| 001 | Sino Biological | Rabbit | IgG* | Recombinant human CD44 |  |
| 02 | Sino Biological | Mouse | IgG1 | Recombinant human CD44 |  |
| 065 | Sino Biological | Rabbit | IgG | Recombinant human CD44 |  |
| 1 | Invitrogen | Rabbit | IgG | Recombinant human CD44 |  |
| 10D1 | LSBio | Rat | IgG1 | Recombinant CD44v4** fusion protein |  |
| 10E13 | US Biologicals | Mouse | IgG1 | Recombinant CD44. |  |
| 113 | Sino Biological | Rabbit | IgG | Recombinant human endothelial cell adhesion molecule |  |
| 13B135 | US Biologicals | Mouse | IgG1 | Recombinant fragment of human CD44 (aa^#^ 628-699) expressed in *E. coli*. |  |
| 156-3C11 | Abcam, Abeomics, Abnova, Bio-Rad, Cell Signaling, ImmuQuest; LSBio; Novus Biologicals; Santa Cruz Biotechnology | Mouse | IgG2a | Stimulated human leukocytes and recognizes residues surrounding proline 210 of human CD44 |  |
| 15C6 | LSBio | Mouse | IgG2a kappa | CD44 of MML-1 human leukemia cells |  |
| 19H8L4 | Invitrogen | Rabbit | IgG | Peptide corresponding to aa 411–424 of human CD44 |  |
| 1B12 | Abnova | Mouse | IgG2a kappa | CD44 aa 699 |  |
| 1C11H1 | Sino Biological | Mouse | IgG1 | Recombinant Human CD44 |  |
| 1E1 | Abnova | Mouse | IgG1 | CD44 aa 699 |  |
| 1F10/B8 | LSBio | Mouse | IgG1 kappa | CD44 of purified human small basement membrane heparan sulfate proteoglycan |  |
| 1G4 | Abnova | Mouse | IgG2a kappa | CD44 aa 699 |  |
| 1M7.8.1 | Abcam, GeneTex; Invitrogen, Novus Biologicals; Creative Biolabs | Rat | IgG2b | Full length protein corresponding to mouse CD44 |  |
| 289-16211 | Abnova | Mouse | IgG2a, kappa | Native purified CD44 from ocular melanoma cell line |  |
| 2C5 | R&D Systems | Mouse | IgG2a | All CD44 isoforms |  |
| 2F10 | R&D Systems | Mouse | IgG1 | Recombinant human CD44 v3-10 |  |
| 2H5 | Abnova | Mouse | IgG1 kappa | CD44 aa 699 |  |
| 2H7/G11 | LSBio | Mouse | IgG1 kappa | Purified human small basement membrane heparan sulfate proteoglycan. |  |
| 2Q1374 | MyBioSource | Rat | IgG2b | CD44 isoforms |  |
| 32 | Abnova | Mouse | IgG1 | Recombinant protein corresponding to full length human CD44. |  |
| 3E8C3 | Sino Biological | Mouse | IgG1 | Recombinant human CD44 |  |
| 3G5 | R&D Systems | Mouse | IgG2b | Recombinant human CD44v3-10 |  |
| 42 | Abnova | Mouse | IgG1 | Recombinant protein corresponding to full length human CD44. |  |
| 4D2 | Abnova | Mouse | IgG2a kappa | CD44 aa 699 |  |
| 4H2 | Abnova | Mouse | IgG2a kappa | CD44 aa 699 |  |
| 5035-41.1D | LSBio | Mouse | IgG1 | Spleen cells from B6PL-Ly-2a / Ly-3a B6-Ly-1a mice. |  |
| 515 | BD Bioscience | Mouse | IgG1 kappa | 80-95 kDa CD44 |  |
| 5C10 | Abnova Antibodies-online; LSBio; MyBioSource, NKMAX; | Mouse | gG2b, kappa | The aa 21-145 of human CD44 |  |
| 5F12 | LSBio | Mouse | IgG1 | EBV transformed normal human B-lymphocytes. |  |
| 65 | Invitrogen | Rabbit | IgG | Recombinant human CD44 protein |  |
| 691534 | R&D Systems | Mouse | IgG2a | Mouse myeloma cell line NS0-derived recombinant human CD44, aa 21-220 |  |
| 6D33 | MyBioSource | Mouse | IgG1 | Exon v7 CD44. |  |
| 6F4H2 | Proteintech | Mouse | IgG2a | CD44 |  |
| 7F1 | Invitrogen | Mouse | IgG1 | CD44 of TCR-gamma epsilon thymocytes |  |
| 8E2 | Cell Signaling | Mouse | IgG1 | CD44 |  |
| 8E2F3 | Abnova; Invitrogen; LSBio, MyBioSource; OriGene; ProMab | Mouse | IgG1 | Recombinant protein corresponding to aa 628-699 of human CD44 |  |
| 9A4 | LSBio | Rat | IgG1 | CD44 |  |
| A020 | EMD Millipore | Rat | IgG2b | Human lymphocyte CD44 |  |
| A3D8 | Millipore Sigma | Mouse | IgG1 | Circulating malignant human Sezary T cells |  |
| ABN-13 | Abnova | Mouse | IgG2a | CD44 |  |
| AGI-3 | Booster | Rabbit | IgG | A synthesized peptide derived from human CD44 |  |
| AV6 | LSBio | Mouse | IgG1 | CD44 |  |
| B-F24 | MyBioSource; Santa Cruz Biotchnology | Mouse | IgG1 kappa | CD44 85-250 kDa |  |
| B313 | LSBio | Mouse | IgG1 kappa | CD44 |  |
| BJ18 | BioLegend | Mouse | IgG  kappa | CD44 |  |
| BRIC 222 | American Research Prodcuts; International Blood Group Reference Laboratory | Mouse | IgG1  kappa | 80 kDa CD44 |  |
| BRIC 235 | American Research Products; International Blood Group Reference Laboratory | Mouse | IgG2b kappa | 80 kDa CD44. |  |
| BSB-12 | LSBio | Mouse | IgG2a | CD44 |  |
| BU52 | Abnova, Bio-Rad; Invitrogen, LSBio; Sanat Cruz Biotechnology | Mouse | IgG1 | Native CD44 from human peripheral myeloma cell |  |
| Bu75 | LSBio | Mouse | IgG2a | Human CD44 from peripheral myeloma cells |  |
| CD44v4/1219 | Abcam, Abnova; Novus Biologicals | Mouse | IgG2a kappa | Recombinant fragment corresponding to the v4 domain of human CD44 |  |
| CD44v4/1700R | Abcam | Rabbit | IgG Kappa | Recombinant fragment corresponding to the v4 domain of human CD44 |  |
| CD44v6/1246 | Abcam; Abnova; Novus Biologicals | Mouse | IgG2a kappa | Recombinant fragment corresponding to human  v6 |  |
| CD44v9/1459 | Abnova, Novus Biologicals; | Mouse | IgG1, Kappa | Recombinant protein corresponding to human CD44v9 |  |
| DF1485 | Dako (Agilent); Santa Cruz Biotechnology, Abnova | Mouse | IgG1 kappa | CD44 |  |
| EPR1013Y | Abcam | Rabbit | IgG | Synthetic peptide human CD44 aa 150-250. |  |
| EPR18668 | Abcam | Rabbit | IgG | Recombinant fragment w human CD44 aa 1-250. |  |
| F-4 | Santa Cruz Biotechnology | Mouse | IgG1 kappa | The aa 21-320 of CD44 |  |
| F10-44-2 | Abcam, Bio-Rad; EMD Millipore; Invitrogen; LSBio; Novus Biologicals; Proteintech; Santa Cruz Biotechnology | Mouse | IgG2a | CD44 from human T lymphocytes |  |
| FNab01483 | Wuhan Fine Biotech Co.,Ltd. | Mouse | IgG2a | CD44 |  |
| fw11-10-3 | ATCC | Mouse | IgG2A | Human CD44;exons v3 to v10 |  |
| G44-26(C-26) | BD Bioscience | Mouse | IgG2b | 80-95 kDa glycosylated CD44 |  |
| GT462 | GeneTex | Mouse | IgG2b | Recombinant protein encompassing a sequence within the center region of human CD44. |  |
| GT981 | GeneTex | Mouse | IgG1 | CD44 80kDa |  |
| hb-256 | ATCC | Mouse | IgG2A | Human CD44 v6 |  |
| hb-258 | ATCC | Mouse | IgG1 | Human CD44 v9 |  |
| HCAM / 918 | Novus Biologicals | Mouse | IgG2a kappa | CD44 80-95kDa |  |
| HCAM/1097 | Abnova | Mouse | IgG1, kappa | Recombinant protein corresponding to full length human CD44. |  |
| HCAM/918 | Abnova | Mouse | IgG2a, kappa | Recombinant protein corresponding to full length human CD44 |  |
| hCD44 | MyBioSource | Mouse | IgG1 | CD44 |  |
| Hermes-1 | Absolute Antibody; Bio X Cell; Invitrogen; LSBio; Novus Biologicals; | Rat | IgG2a | CD44 of human tonsil lymphocytes |  |
| Hermes-3 | Abnova Absolute Antibodies; LSBio; US Biologicals | Mouse | IgG2a, kappa | Recombinant CD44 |  |
| HI44a | Abnova | Mouse | IgG2a | Native CD44 |  |
| IM7 | Abnova, Bio X Cell, Bio-Rad, BioLegend; Exbio, Invitrogen; LSBio; Novus Biologicals; OriGene; R&D Systems; Stem Cell Technologies | Rat | IgG2b, kappa | Human CD44 and mouse CD44 |  |
| IQ3/0 | Creative Biomart | Mouse | IgG1 | CD44 of leukemia T cells |  |
| IRAWB 14.4 | Santa Cruz Biotechnology | Rat | IgG2a | HA-binding variant of a T-cell line derived from a spontaneous tumor of mouse origin. |  |
| J.173 | Beckman Coulter | Mouse | IgG1 | CD44 of LAZ 221 |  |
| KM114 | Santa Cruz Biotechnology | Rat | IgG1 | CD44 of BMS2 bone marrow derived stromal cell line of mouse origin |  |
| KM81 | LSBio; MyBioSource | Rat | IgG2a | Bone marrow derived stromal cells (clone BMS2) |  |
| KZI | International Blood Group Reference Laboratory | Mouse | IgG1 | CD44 of fibroblast |  |
| L178 | BD Bioscience | Mouse | IgG1 kappa | Human TCR γδ+ Thymocytes |  |
| LS-C121538 | LSBio | Mouse | IgG2a | Human T lymphocytes |  |
| LS-C123022 | LSBio | Mouse | IgG1 | Sheep CD44. |  |
| LS-C123024 | LSBio | Mouse | IgG1 | CD44 of rat T cell blasts |  |
| LS-C123025 | LSBio | Mouse | IgG1 | CD44 of RL-5 T cell line |  |
| LS-C123738 | LSBio | Rat | IgG2a kappa | CD44 from EHS mouse tumor. |  |
| LS-C127692 | LSBio | Mouse | IgG1 | Recombinant CD44 |  |
| LS-C13434 | LSBio | Rat | IgG2b | Purified human blood lymphocyte CD44 |  |
| LS-C178793 | LSBio | Mouse | IgG1 | Recombinant protein corresponding to full length CD44v6. |  |
| LS-C178794 | LSBio | Mouse | IgG1 | Recombinant protein corresponding to full length CD44v6. |  |
| M588 | BioLegend | Mouse | IgG kappa | CD44 |  |
| MA54 | Invitrogen | Mouse | IgG1 | Epitope encoded by exon v6 on the variant portion of human CD44 |  |
| MEM-263 | Abnova; Invitrogen; LSBio; Millipore-Sigma | Mouse | IgG1 | Native purified CD44 from african green monkey COS-7 cells |  |
| MEM-263 | Exbio | Mouse | IgG1 | CD44 from MEM-263 cells |  |
| MEM-85 | Abcam; Abnova; Exbio; Invitrogen; LSBio; Millipore-Sigma | Mouse | IgG2b | CD44 from human leukemia cells. |  |
| MRC OX-49 | LSBio, MyBioSource; Santa Cruz Biotechnology | Mouse | IgG2a | CD44 from T cell blasts. |  |
| MRC OX-50 | LSBio | Mouse | IgG1 | CD44 from rat T cell blasts |  |
| MRQ-13 | OriGene | Mouse | IgG2a | CD44 |  |
| NKI-P2 | Santa Cruz Biotechnology | Mouse | IgG1 | Immunoprecipitated CD44. |  |
| OTI1A2 | LSBio; OrigGene | Mouse | IgG1 | Full length human recombinant protein of human CD44 produced in HEK293T cell. |  |
| OTI1C8 | LSBio; OriGene | Mouse | IgG1 | Full length human recombinant protein of human CD44 produced in HEK293T cell. |  |
| OTI1D8 | LSBio; OriGene | Mouse | IgG1 | Full length human recombinant protein of human CD44 produced in HEK293T cell. |  |
| OTI1E3 | LSBio; Novus Biologicals, OriGene | Mouse | IgG1 | Full length human recombinant protein of human CD44 produced in HEK293T cell. |  |
| OTI1G1 | LSBio; OriGene | Mouse | IgG2A | Full length human recombinant protein of human CD44 produced in HEK293T cell. |  |
| OTI1G8 | LSBio; Origene | Mouse | IgG1 | Full length human recombinant protein of human CD44 produced in HEK293T cell. |  |
| OTI2D9 | LSBio; OriGene | Mouse | IgG1 | Full length human recombinant protein of human CD44 produced in HEK293T cell. |  |
| OTI2E3 | LSBio; OriGene | Mouse | IgG1 | Full length human recombinant protein of human CD44 produced in HEK293T cell. |  |
| OTI5B4 | LSBio; OriGene | Mouse | IgG2A | Full length human recombinant protein of human CD44 produced in HEK293T cell. |  |
| OTI5B5 | LSBio; OriGene | Mouse | IgG2A | Full length human recombinant protein of human CD44 produced in HEK293T cell. |  |
| OTI5B6 | LSBio; OriGene | Mouse | IgG2A | Full length human recombinant protein of human CD44 produced in HEK293T cell. |  |
| OTI5B7 | LSBio; OriGene | Mouse | IgG2a | Full length human recombinant protein of human CD44 produced in HEK293T cell. |  |
| OTI5C1 | LSBio; OriGene | Mouse | IgG2A | Full length human recombinant protein of CD44 produced in HEK293T cell. |  |
| OTI5C4 | LSBio; OriGene | Mouse | IgG2A | Full length human recombinant protein of human CD44(NP_000601) produced in HEK293T cell. |  |
| OTI5D5 | LSBio; OriGene | Mouse | IgG2A | Full length human recombinant protein of human CD44 produced in HEK293T cell. |  |
| OTI5E6 | LSBio; OriGene | Mouse | IgG2a | Full length human recombinant protein of human CD44 produced in HEK293T cell. |  |
| P1G12 | Santa Cruz Biotechnology | Mouse | IgG1 kappa | CD44 from HT-1080 fibrosarcoma cells of human origin. |  |
| P2A1 | Santa Cruz Biotechnology | Mouse | IgG2a | CD44 from ocular melanoma cell line of human origin. |  |
| P3H9 | Santa Cruz Biotechnology | Mouse | IgG1 kappa | CD44 from HT-1080 fibrosarcoma cells of human origin. |  |
| REA706 | Miltenyi Biotec | Cell line | IgG1 kappa | CD44v6 |  |
| RM264 | Abnova | Rabbit | IgG | A synthetic peptide corresponding to extracellular domain region of human CD44. |  |
| RV3 | Abnova, BioLegend; Cosmo Bio | Rat | IgG2a | Recombinant human CD44 v8-10. |  |
| SAIC-01B-4D8 | Absolute Antibody | Rabbit | IgG | Peptide "TFIPVTSAK" derived from CD44 |  |
| SFF-2 | Invitrogen; Santa Cruz Biotechnology | Mouse | IgG1, kappa | CD44std |  |
| SFF-304 | Enzo Life Science; Invitrogen | Mouse | IgG1 | Recombinant human CD44 |  |
| SP37 | Abcam Abnova | Rabbit | IgG | Synthetic peptide within human CD44 aa 150-250 |  |
| SPM521 | Abnova | Mouse | IgG2a | A synthetic peptide corresponding to internal region of human CD44. |  |
| SPM544 | Abnova; Novus Biologicals | Mouse | IgG2a, kappa | Native CD44 |  |
| UMAB133 | OriGene | Mouse | IgG1 | CD44 |  |
| VFF-11 | Antibodies-online, Bio-Rad; Invitrogen, LSBio | Mouse | IgG1 | Fusion protein corresponding to CD44ExonV4 |  |
| VFF-14 | Bio-Rad; Enzo Life Science; Invitrogen; LSBio | Mouse | IgG1 | Fusion protein corresponding to CD44v10 |  |
| VFF-17 | Bio-Rad, Invitrogen; LSBio | Mouse | IgG2b | Fusion protein corresponding to CD44v7/8 |  |
| VFF-18 | Abcam, Bio-Rad; EMD Millipore, Invitrogen | Mouse | IgG1 | Fusion protein corresponding to Human  V 6 |  |
| VFF-327V3 | Abcam, Bio-Rad; Invitrogen; LSBio | Mouse | IgG1 | Fusion protein corresponding to human v3 |  |
| VFF-7 | Antibodies-online, Bio-Rad; Invitrogen; LSBio; Santa Cruz Biotechnology | Mouse | IgG1 | Fusion protein corresponding to ExonV6 |  |
| VFF-8 | Bio-Rad; Invitrogen; LSBio | Mouse | IgG1 | Fusion protein corresponding to Exonv5 |  |
| VFF-9 | Bio-Rad; Enzo Life Science; Invitrogen, LSBio | Mouse | IgG1 | Fusion protein corresponding to Exonv7 |  |
| VMA00034 | Bio-Rad | Mouse | IgG2a | CD44 aa 742 |  |
| VMA00034KT | Bio-Rad | Mouse | IgG2a | CD44 aa 742 |  |
