# Supplementary material for: New monoclonal antibodies that recognize an unglycosylated, conserved, extracellular region of CD44 in vitro and in vivo, and can block tumorigenesis
Source: PLoS One. 2021 Apr 23;16(4):e0250175. doi: 10.1371/journal.pone.0250175 (PMC8064539; doi:10.1371/journal.pone.0250175)
Supplement: S2 Table — (DOCX) [file pone.0250175.s002.docx]

S2 Table: primer sequences and location of binding.

Primer applied in the RT-PCR and their location on the coding region of CD44isf12 and GAPDH, respectively; Fw, forward primer; Rv, reverser primer; Bp, base pair;

| Primer | Sequence | Location (Bp) |
| --- | --- | --- |
| CD44Fw | 5’-ATGGACAAGTTTTGGTGG  CACGCAGCCTGGGGACTCTGCCTCG-3’ | 1-43 |
| CD44Rv | 5’-TTACACCCCAATCTTCATGTCCACATTCTGC-3’ | 1490-1520 |
| GAPDHFw | 5’-CATCTTCTTTTACATCGCC-3’ | 99-118 |
| GAPDHRv | 5’-GAGACCACCTGGTGCTCAGTGTAG-3’ | 699-722 |
