# Supplementary material for: New monoclonal antibodies that recognize an unglycosylated, conserved, extracellular region of CD44 in vitro and in vivo, and can block tumorigenesis
Source: PLoS One. 2021 Apr 23;16(4):e0250175. doi: 10.1371/journal.pone.0250175 (PMC8064539; doi:10.1371/journal.pone.0250175)
Supplement: S1 Raw images — (PDF) [file pone.0250175.s003.pdf]

Raw Images for Fig. 3A

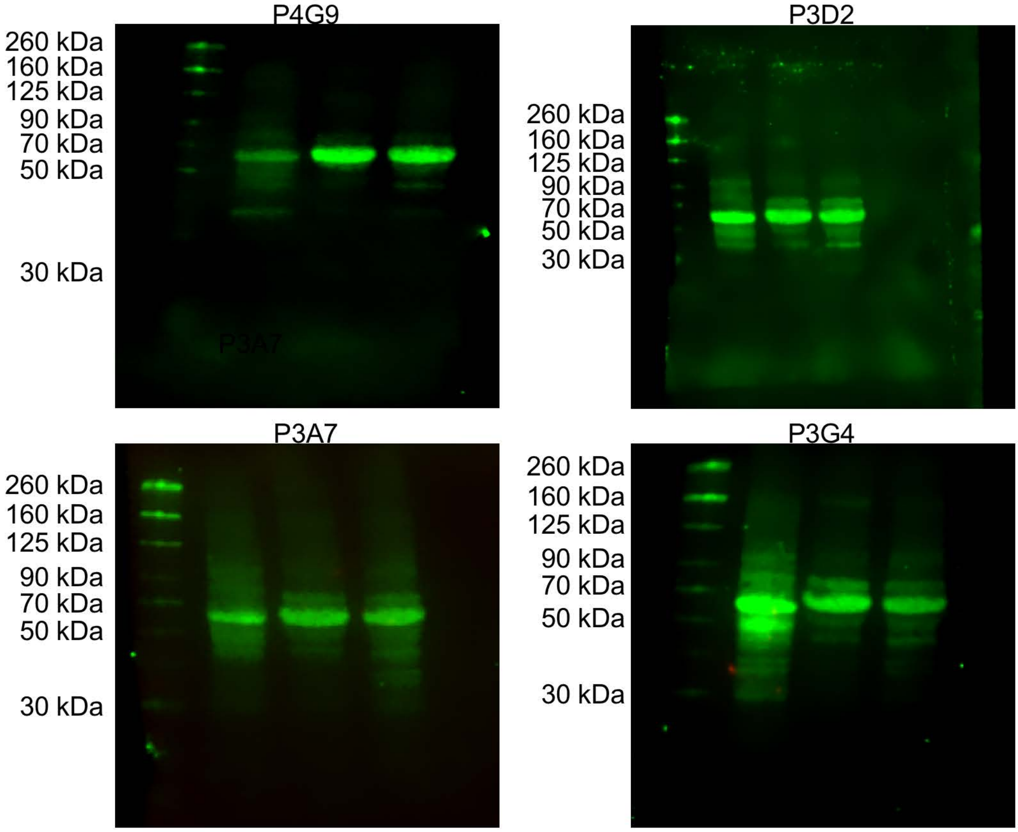

Raw Images for Fig. 3B

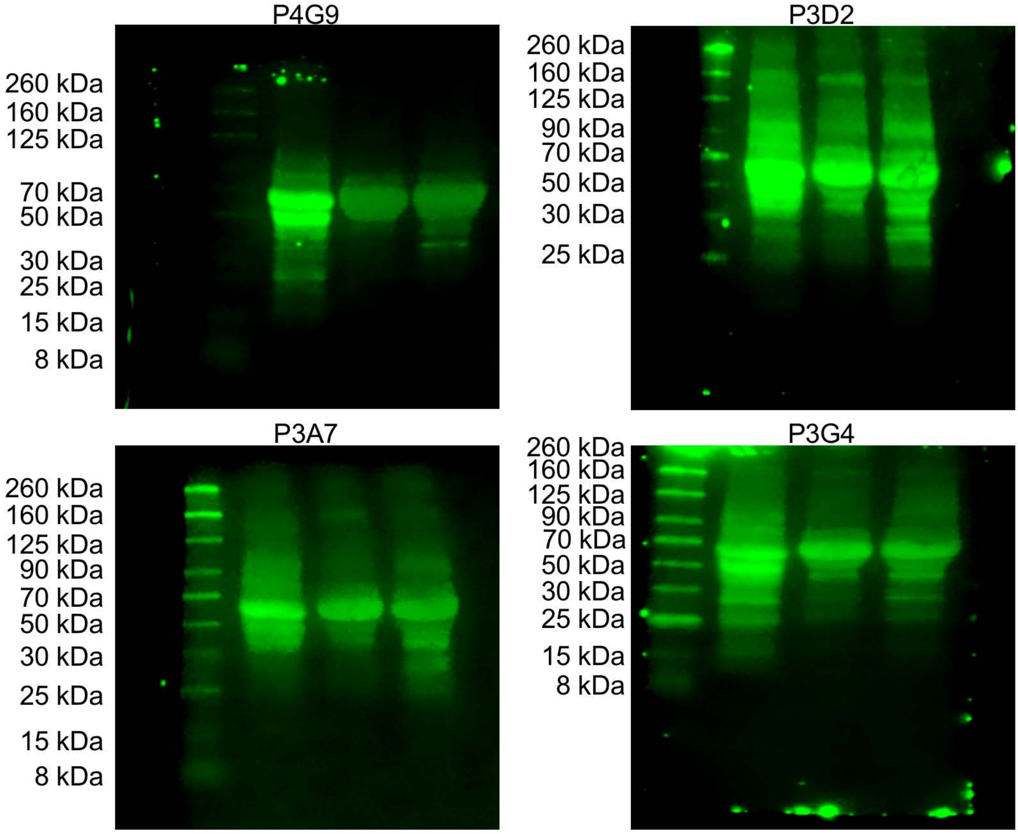

In all experiments the marker was loaded first, followed by the lysate of MB-231, HTB-66 and U87 cells. The whole range of the marker is shown. Scanning was done using a Li-COR Odyseey FC.
For Fig.3A and B the gels have been cropped, the image was converted to gray scale, inverted and contrast enhanced to show mAb binding to proteins in the lysate at low and high amount of lysate

Raw Images for Fig. 4C

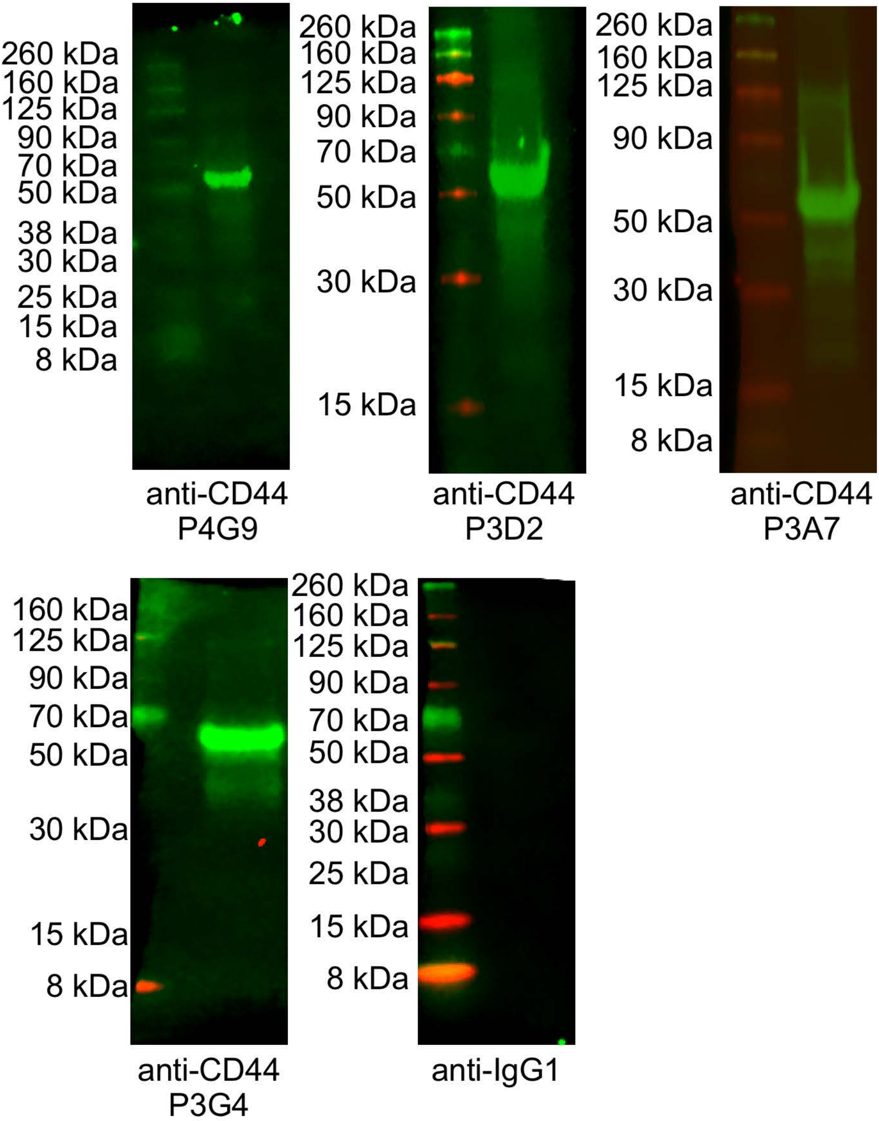

The marker was loaded first, then the recombinant protein p2.
The whole range of the marker is shown. Scanning was done using a Li-COR Odyseey FC.
For Fig. 4C the bands were cropped to the area between 50 kDa and 70 kDa,
the image converted to gray scale, inverted and contrast enhanced to show mAb binding
to the recombinant protein

Raw Images for Fig. 5A.

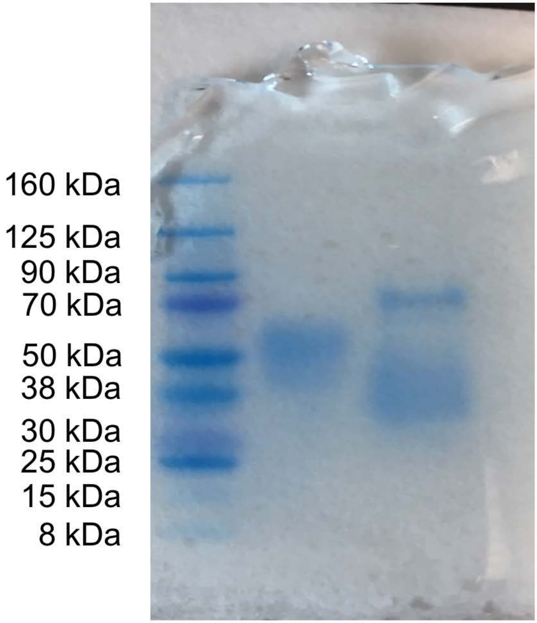

The marker was loaded first, then the control sample containing recombinant protein p4, followed by the sample that contains the recombinant protein p4 and glycosidases.The whole range of the marker is shown. The gel was Coomassie stained.

For Figur 5 the gel was cropped to the area that shows the shift of the recombinant protein p4.

The image was converted to gray scale, inverted and contrast enhanced.
